# A KDM6 inhibitor potently induces ATF4 and its target gene expression through HRI activation and by UTX inhibition

**DOI:** 10.1038/s41598-021-83857-y

**Published:** 2021-02-25

**Authors:** Shojiro Kitajima, Wendi Sun, Kian Leong Lee, Jolene Caifeng Ho, Seiichi Oyadomari, Takashi Okamoto, Hisao Masai, Lorenz Poellinger, Hiroyuki Kato

**Affiliations:** 1 Cancer Science Institute of Singapore, National University of Singapore, 14 Medical Drive, Singapore, 117599 Republic of Singapore; 2 Institute for Advanced Biosciences, Keio University, Kakuganji 246-2, Mizukami, Tsuruoka, Yamagata 997-0052 Japan; 3 Cancer & Stem Cell Biology Programme, Duke-NUS Medical School, 8 College Road, Singapore, 169857 Republic of Singapore; 4 Institute of Advanced Medical Sciences, Tokushima University, Tokushima, 770-8503 Japan; 5 Department of Molecular and Cellular Biology, Nagoya City University Graduate School of Medical Science, Mizuho-ku, Nagoya, 467-8601 Japan; 6 Genome Dynamics Project, Department of Basic Medical Sciences, Tokyo Metropolitan Institute of Medical Science, 2-1-6 Kamikitazawa, Setagaya-ku, Tokyo 156-8506 Japan; 7 Department of Cell and Molecular Biology, Karolinska Institutet, 171 77 Stockholm, Sweden

**Keywords:** Biochemistry, Cancer, Cell biology, Drug discovery, Molecular biology

## Abstract

*UTX/KDM6A* encodes a major histone H3 lysine 27 (H3K27) demethylase, and is frequently mutated in various types of human cancers. Although UTX appears to play a crucial role in oncogenesis, the mechanisms involved are still largely unknown. Here we show that a specific pharmacological inhibitor of H3K27 demethylases, GSK-J4, induces the expression of transcription activating factor 4 (ATF4) protein as well as the ATF4 target genes (e.g. *PCK2, CHOP, REDD1, CHAC1* and *TRIB3*). ATF4 induction by GSK-J4 was due to neither transcriptional nor post-translational regulation. In support of this view, the ATF4 induction was almost exclusively dependent on the heme-regulated eIF2α kinase (HRI) in mouse embryonic fibroblasts (MEFs). Gene expression profiles with *UTX* disruption by CRISPR-Cas9 editing and the following stable re-expression of UTX showed that UTX specifically suppresses the expression of the ATF4 target genes, suggesting that UTX inhibition is at least partially responsible for the ATF4 induction. Apoptosis induction by GSK-J4 was partially and cell-type specifically correlated with the activation of ATF4-CHOP. These findings highlight that the anti-cancer drug candidate GSK-J4 strongly induces ATF4 and its target genes via HRI activation and raise a possibility that UTX might modulate cancer formation by regulating the HRI-ATF4 axis.

## Introduction

UTX (Ubiquitously Transcribed Tetratricopeptide Repeat on X Chromosome), also referred to as lysine demethylase 6A (KDM6A), is a major H3K27 demethylase^1–4^. It erases the transcriptional repressive mark (tri-methylated H3K27) and thereby activating the expression of target genes. UTX forms multi-protein complexes with the mixed-lineage leukemia H3K4 methyltransferases (MLL3/4)^5–7^ to cooperatively and simultaneously function with each other^8,9^, and counteracts the polycomb complex (PRC2) that has the H3K27 methyltransferase activity^10,11^. UTX has also been reported to play essential roles in various biological processes such as embryonic development, cell differentiation, and stem cell maintenance^12^.

*UTX* mutations have also been found in various types of human cancers: clear cell renal cell carcinoma, medulloblastoma, chronic myelomonocytic leukemia, acute lymphoblastic leukemia, transitional cell carcinoma of the bladder, and high-grade muscle-invasive urothelial bladder carcinoma and others^12,13^. UTX and the other major H3K27 demethylase JMJD3 (KDM6B) have been reported to play major roles in cell proliferation and malignancy in several different experimental systems. UTX was reported to regulate expression of RB and RB-binding proteins by directly binding to their promoters and cause cell cycle arrest in primary human fibroblasts^14^. UTX negatively regulates proliferation, clonogenicity, adhesion and xenograft tumorigenicity of multiple myeloma^15^. Jmjd3, but not Utx, is induced by the Ras-Raf signaling and activates p16Ink4a and p19Arf to suppress cell proliferation in mouse embryonic fibroblasts^16,17^. In the Notch1-induced T-cell acute lymphoblastic leukemia (T-ALL) mouse model, Notch1 increases Jmjd3 expression likely via NFkB, which in turn enhances the expression of Notch1 target genes in cooperation with Notch1, while Utx suppresses the T-ALL formation^18^. Somewhat paradoxically, however, knockdown of UTX suppresses proliferation of breast cancer cell lines in vitro and the invasiveness of their xenografts in mice^19^, and UTX promotes the proliferation and migration of estrogen receptor-positive breast cancer cells^20^. Thus the contribution of UTX and JMJD3 to cancer formation and progression appears to be highly context-dependent, which remains to be mechanistically and comprehensively explained.

GSK-J1 is a potent KDM6 (both JMJD3 and UTX) inhibitor in in vitro binding and enzyme assays^21^. GSK-J4 is the cell-permeable form of, and is converted to, GSK-J1 and inhibits TNFα production in human primary macrophages^21^. GSK-J4 has been considered as a therapeutic tool against various malignancies^22^ such as T-ALL, pediatric brainstem glioma, diffuse intrinsic pontine glioma, non-small cell lung cancer, breast cancer stem cells, castration-resistant prostate cancer, and high-risk neuroblastoma. However, these studies mainly focused on inhibitory effects on cell proliferation, and the molecular mechanisms involved in these therapeutic effects remain poorly understood. In a subsequent study, also using an in vitro inhibition and cell-based assays, GSK-J4 significantly inhibited other KDMs such as KDM5B and KDM5C^23^. While this discrepancy in target specificity could be due to difference in intracellular conversion rates from GSK-J4 to GSK-J1^24^, further studies on the GSK-J4 specificity are required.

To gain insights into UTX functions for cancer formation and maintenance, we utilized the KDM6 inhibitor, GSK-J4, and UTX-deficient cells created using the CRISPR-Cas9 editing system in conjunction with complementation with exogenous UTX. We found that ATF4 and its downstream target genes are highly and rapidly induced by GSK-J4 independently of the canonical ER stress response and that one of the integrated stress response (ISR) kinases, HRI, is mainly responsible for the ATF4 induction. Expression of the ATF4 target genes was specifically suppressed by UTX complementation. It is unlikely that activation of the ATF4-CHOP cascade by GSK-J4 is a sole or major determinant of apoptosis induction. These results revealed a unique physiological effect of GSK-J4 and provide insights into therapeutic use of KDM6 inhibitors.

## Results

### GSK-J4 induces expression of ATF4 and its target genes independently of the unfolded protein response (UPR)

**Figure 1:**
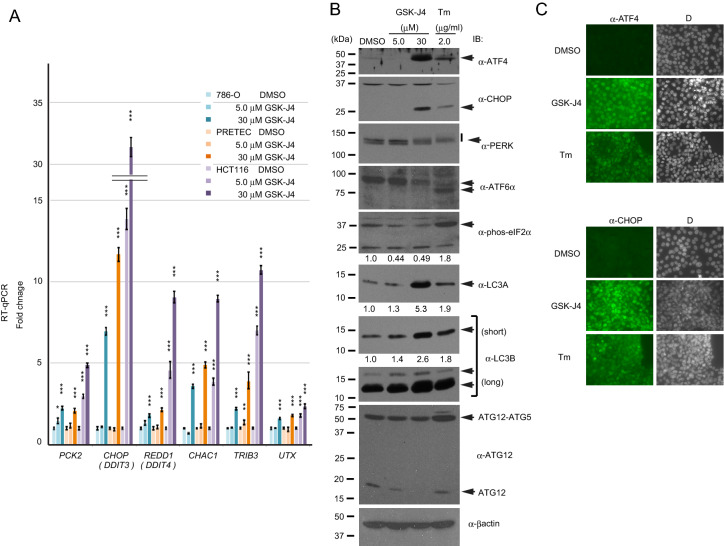
GSK-J4 induces the expression of ATF4 protein and its downstream target genes. (**A**) Analysis of GSK-J4-regulated gene expression by reverse transcription-quantitative PCR (RT-qPCR). Cells (786-O, PRETEC and HCT116) were treated with the indicated concentrations (0, 5 or 30 μM) of GSK-J4 for 20 h and their isolated total RNA was analyzed. Bars show the mean of biological triplicates (N = 3) with standard error of the mean (S.E.M.) bars and p-value asterisks (*< 0.05; **< 0.01; ***< 0.001). Asterisks are placed vertically. Fold changes were calculated against the DMSO control of each cell type at 1.0 after normalizing against the PPIA housekeeping gene. Primer sequences are presented in Table S1. (**B**) Western blot analysis. Total cell extracts from HCT116 treated with GSK-J4 or tunicamycin (Tm) for 20 h at the indicated concentrations were analyzed with the antibodies denoted on the right. Arrows indicate specific bands. Vertical short bar indicates the phosphorylated (sifted up) PERK band. Bands were quantified and relative values to the control (DMSO) are presented. Both short and long exposure photos are presented for LC3B. (**C**) Similarly treated HCT116 cells were also analyzed by immunofluorescence (IF) staining with antibodies against ATF4 or CHOP. Fluorescence images after staining with Alexa 488-conjugated secondary antibody and DAPI (**D**) are shown. Staining was performed under the same conditions. Images were captured by the exposure of 1000 ms for immunostaining and 80 ms for DAPI.

We have found that GSK-J4 treatment up-regulated the mRNA expression levels of genes targeted by ATF4 (*PCK2, CHOP (DDIT3), REDD1 (DDIT4), CHAC1*, and *TRIB3*)^25–29^, with minimal changes to *UTX* expression, in epithelial cells: clear cell renal cell carcinoma (ccRCC) 786-O cells, primary renal proximal tubule epithelial cells (PRETEC) and colorectal cancer HCT116 cells (Fig. 1A). The induction of the ATF4 target genes by GSK-J4 was high especially in HCT116. As one of the three branches of the UPR (or ER stress response), the protein kinase R-like ER kinase (PERK) undergoes autophosphorylation and phosphorylates the eukaryotic translation initiation factor 2 subunit 1α (eIF2α) at Ser51 and thereby protein synthesis is attenuated^30^. Functional ATF4 is then produced by overriding the upstream abortive initiation sites and activates the downstream target genes^31^. Treatment with an ER stress inducer, tunicamycin (Tm), led to the typical UPR (Fig. 1B): PERK and eIF2α were phosphorylated^30^; ATF4 and its downstream target CHOP, a C/EBP family transcription factor, were induced^31^; ATF6α was cleaved by proteolysis^32^; the autophagy-related components (LC3A and LC3B) were activated, and ATG5-ATG12 conjugation occurred^33,34^. On the other hand, GSK-J4 minimally enhanced phosphorylation of PERK and eIF2α under this condition (Fig. 1B). Induction of the nuclear localizing ATF4 and CHOP proteins was confirmed by immunofluorescence (IF) staining of HCT116 cells treated with GSK-J4 or Tm (Fig. 1C). Thus, it is unlikely that the induction of ATF4 and CHOP by GSK-J4 proceeds through the canonical UPR pathway.

### GSK-J4 induction of ATF4 is early, potent and cell type-specific

**Figure 2:**
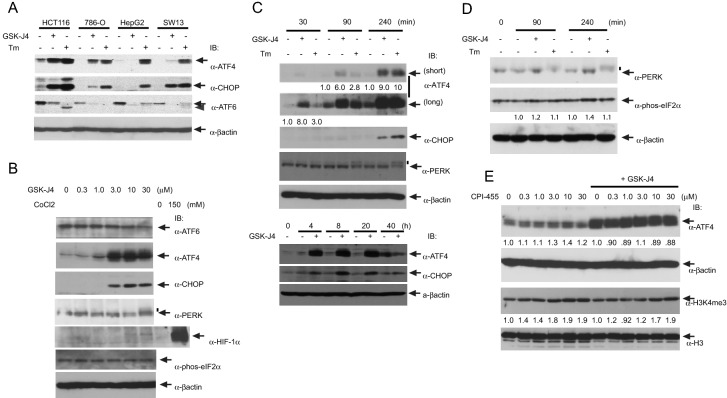
Characterization of GSK-J4 induction of ATF4. (**A**) Cell type specificity. Four epithelial cancer cell lines were tested for induction of ATF4, ATF6α and CHOP at 30 μM GSK-J4 or 2.0 μg/ml Tm for 20 h. Specific, unmodified bands are indicated by arrows and the processed ATF6α band by an arrowhead. Note that in HCT116, ATF4 and CHOP were highly induced by both Tm and GSK-J4, whereas ATF6α processing was induced by Tm but not by GSK-J4. (**B**) Concentration-dependent changes in protein expression and modification in HCT116. To increase the sensitivity to GSK-J4, this experiment was performed at lower cell confluency (20% at GSK-J4 administration). The vertical short bar indicates phosphorylated (upshifted) PERK bands. (**C**) Time course of protein induction by GSK-J4. Induction was examined over a short (upper panel) and long (lower panel) duration as indicated. ATF4 bands were quantified and their values are presented under the lanes. (**D**) Short time course experiment for detection of eIF2α phosphorylation. (**E**) Effect of the KDM5 inhibitor CPI-455 on ATF4 induction in HCT116. Cells were first treated with the indicated concentration of CPI-455 for 20 h and then incubated for another 20 h in the absence or presence of 30 μM GSK-J4. Quantified band intensities are presented under the lanes.

The cell type specificity of the GSK-J4 effect on ATF4 and CHOP expression was further tested. HCT116, 786-O and SW13 efficiently responded to GSK-J4 but HepG2 did not (Fig. 2A), whereas all the cell lines responded to Tm. Because of its most robust response, HCT116 was used for the following experiments. GSK-J4 strongly induced ATF4 and CHOP at concentrations of 3.0, 10 and 30 μM (Fig. 2B). According to the previous reports, GSK-J4 inhibits lipopolysaccharide-induced TNFα production in human primary macrophages with IC_50_ of 9 µM^21^ and blocks growth of T cell acute lymphoblastic leukemia (T-ALL) cells with IC_50_ of 2 μM (25), indicating that GSK-J4 is a highly potent inducer of ATF4 and CHOP. PERK phosphorylation, detected as an upshift in the PERK band, was observed slightly at the highest concentration (30 μM) of GSK-J4, although eIF2α phosphorylation was unchanged (Fig. 2B), in agreement with the results seen in Fig. 1B. GSK-J4 is an α-ketoglutarate (α-KG) antagonist^21^ which could therefore act as an inhibitor of the prolyl hydroxylase domain proteins (PHDs) that hydroxylate and destabilize hypoxia-inducible factors (HIF-1α and HIF-2α) in an α-KG dependent reaction^35,36^. PHD3 reportedly destabilizes ATF4 by hydroxylating several clustered proline residues of ATF4^37^. This raises the possibility that ATF4 stabilization by GSK-J4 could be due to PHD inhibition. At high concentrations of GSK-J4 (10 and 30 μM), HIF-1α was slightly increased to a level much lower than that by the PHD inhibitor cobalt chloride (CoCl_2_) (Fig. 2B), indicating that ATF4 stabilization was not due to PHD inhibition. In a time course experiment, the response to GSK-J4 started at 30 min, which appeared to be faster than that to Tm (Fig. 2C, upper panel). The effect persisted from 4 to 20 h post treatment (Fig. 2C, lower panel). Phosphorylation of PERK by GSK-J4 was also not detected in this experiment (Fig. 2C, upper panel). A slight increase of eIF2α phosphorylation was detected also within the short time period (1.4-fold in 240 min) (Fig. 2D).

Given the concerns over the specificity of GSK-J4^23^, the effect of the KDM5-specific inhibitor CPI-455 on ATF4 expression was tested. In a previous report, 6.25 µM CPI-455 treatment for 5 days increased global trimethylation levels of histone H3 lysine 4 (H3K4me3) in several cancer cell lines and suppressed the growth^38^. After treatment of HCT116 with CPI-455 for 40 h, no increase in ATF4 levels was detected with or without GSK-J4, but a moderate enhancement (up to 1.9-fold) in global H3K4me3 level was observed (Fig. 2E) as previously reported^38^. Therefore, it is unlikely that the induction of ATF4 and CHOP was due to inhibition of the enzymatic activities of the KDM5 family proteins.

### ATF4 induction by GSK-J4 is not primarily due to transcriptional or post-translational activation.

**Figure 3:**
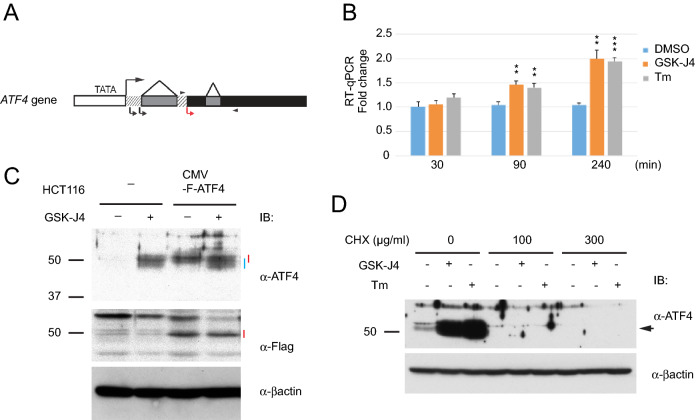
ATF4 induction is not due to an increase in *ATF4* mRNA or ATF4 protein stability. (**A**) Structure of the human *ATF4* gene. Large and small arrows indicate transcription and translation start sites, respectively. Functional ATF4 protein is translated from the small red arrow. Black, grey and slashed boxes indicate coding, intron and 5′ non-coding sequences, respectively. Arrowheads indicate positions of the primers used for RT-qPCR. (**B**) RT-qPCR analysis. Total RNA from HCT116 treated with 30 µM GSK-J4 or 2 µg/ml Tm for 30, 90 or 240 min were analyzed by RT-qPCR with biological triplicates (N = 3). Values were normalized against *PPIA* mRNA. Bars and asterisks indicate S.E.M. and significance, respectively, as in Fig. 1A. (**C**) CMV promoter-driven F-ATF4 was expressed from a lentivirus vector in HCT116. After 4 h exposure, the culture medium was replaced with fresh medium with or without 30 μM GSK-J4 and cultured for 20 h. ATF4 proteins were detected with α-ATF4 (upper panel) or α-Flag antibodies (middle panel). Endogenous and exogenous ATF4 are indicated by blue and red bars, respectively. (**D**) Effect of protein synthesis inhibition. HCT116 cells were treated at the indicated concentrations of cycloheximide (CHX) for 1 h prior to incubation with 30 μM GSK-J4 or 2 µg/ml Tm for 4 h.

Apart from the translational regulation from the downstream initiation site (Fig. 3A)^31^, ATF4 is also regulated at the transcriptional level independently of ER stress. The shorter isoform of CCAAT/enhancer-binding protein ß (LIP) represses ATF4 transcription by targeting its upstream elements^39^. Therefore, we tested this possibility by RT-qPCR analysis. *ATF4* mRNA levels increased by 2.0-fold at 240 min after GSK-J4 administration (Fig. 3B), but ATF4 protein levels were reproducibly increased to a much greater degree (Fig. 2C). Tm also modestly increased *ATF4* mRNA levels (Fig. 3B), as was found in a previous report^31^. These modest increases are most likely due to mRNA stabilization by active translation. Thus, the changes in *ATF4* mRNA levels do not account for the remarkable induction of ATF4 protein by GSK-J4 and it is very unlikely that histone modifications for transcriptional regulation are involved in this ATF4 induction.

ATF4 can be further regulated at the post-translational level. The SCFß-TrCP E3-ligase poly-ubiquitylates ATF4 in a phosphorylation-dependent manner and leads to proteasomal degradation^40^. To analyze ATF4 protein stability, we tested a flag-tagged ATF4 (F-ATF4) fusion protein transcribed from the CMV promoter. Levels of stably expressed F-ATF4, with a slightly higher molecular weight due to the tag, were not increased by GSK-J4, whereas endogenous ATF4 was clearly induced (Fig. 3C). The effect of GSK-J4 was further tested by translational shut down with cycloheximide (CHX). CHX at 100 or 300 µg/ml efficiently suppressed ATF4 induction by GSK-J4, as well as Tm (Fig. 3D). These results indicate that ATF4 induction by GSK-J4 is not due to changes in protein stability. Taken together, increase in the ATF4 protein level can be due to translational regulation.

### ATF4 induction by GSK-J4 depends on HRI

ATF4 is induced also by other stresses than ER stress^41^. In addition to PERK, other kinases also phosphorylate eIF2α Ser51 and induce ATF4 in response to various stresses: collectively named integrated stress response (ISR)^42^. Three other ISR kinases have been identified: GCN2 that responds to amino acid deficiency, HRI (heme-regulated eIF2α kinase) that is activated by heme deprivation in erythroid cells, and PKR (protein kinase R) that is activated by virus infection and other stresses.

**Figure 4:**
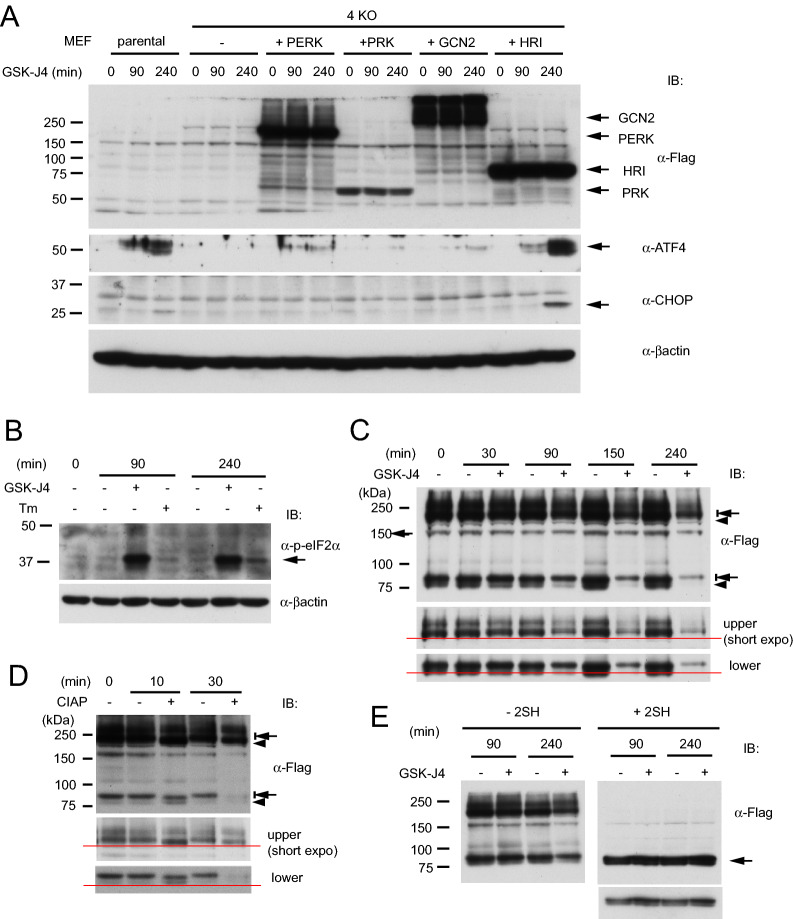
Determination of ISR kinase requirement for induction of ATF4 and CHOP by GSK-J4, and assessment of HRI modification. (**A**) Requirement of HRI for ATF4 and CHOP induction. MEFs lacking expression of all the four ISR kinases (4KO) and expressing one of the kinases by exogenous complementation (+ PERK, + PRK, + GCN2 or + HRI) were tested for their ability to induce ATF4 and CHOP by 30 µM GSK-J4 treatment for 0, 90 or 240 min. (**B**) Phosphorylation of eIF2α. MEFs expressing HRI (MEF-4KO + HRI) were treated with GSK-J4 and analyzed by western blotting. (**C**) HRI migration change in gel. Protein samples from MEF-4KO + HRI were treated with SDS-PAGE sample buffer with no reducing agent for 5 min at 40 °C. Upper and lower band area were dissected and shown below with horizontal red lines to compare migration. Note that GSK-J4-treated HRI bands migrate more slowly or were shifted up. (**D**) Treatment with phosphatase. Extracts from MEF-4KO + HRI treated with GSK-J4 for 90 min were further treated in vitro with CIAP for 10 or 30 min at 30 °C and analyzed by western blotting. *E,* Disruption of high molecular weight bands by a reducing reagent. Extracts from GSK-J4-treated MEF-4KO + HRI were treated with SDS PAGE sample buffer containing no (left panel) or 1% 2-mercaptoethanol (right panel) for 5 min at 40 °C.

We next examined if any of the ISR kinases is required for the GSK-J4-triggered ATF4 induction with the use of mouse embryonic fibroblast cell lines (MEFs) expressing only one of the four kinases^43^. The parental MEF showed efficient ATF4 induction, while MEF with all the four kinase genes inactivated by CRISPR gene editing (MEF-4KO) completely failed (Fig. 4A). Exogenously expressed HRI, which is introduced by a retrovirus vector^43^, restored efficient ATF4 and CHOP induction, whereas other ISR kinases did not (Fig. 4A). GSK-J4 activated eIF2α phosphorylation in MEF-4KO + HRI more strongly than Tm (Fig. 4B), in contrast to the observation with HCT116 (Fig. 2B). Heme depletion causes autophosphorylation of HRI on multiple serine and threonine residues and the extensive phosphorylated forms migrate with retarded mobility in gel^44,45^. HRI is present as multiple dimer forms and an inactive form has an intermolecular disulfide bond^46^. In our time course experiment of GSK-J4 treatment, both high molecular weight (putative dimers) bands and lower bands (putative monomers) of HRI started to shift up mobility on gel as early as in 30 min (Fig. 4C). Overall HRI signals gradually decreased, suggesting that HRI after GSK-J4 treatment is less stable. Consistently, this was also observed for endogenous human HRI in HCT116, HepG2 and SW13 (Supplementary Fig. S1A). Exogenously expressed HRI in HCT116 and HepG2 was strongly stabilized by MG132 (Supplementary Fig. S1B) even in the presence of GSK-J4. This suggests that HRI is subject to proteasome degradation especially after activated by GSK-J4, which could mean presence of a negative feedback regulation to ensure acute and less durable HRI activation.

The extract prepared from cells treated with GSK-J4 for 90 min was further treated in vitro with calf intestinal alkaline phosphatase (CIAP) for 10 min or 30 min (Fig. 4D). The shifted up bands returned to the lower positions, suggesting that HRI was phosphorylated by GSK-J4 treatment^44,45^. To confirm the intermolecular disulfide crosslink of HRI, extracts were treated with 2-mercaptoethanol. As a result, the majority of extracts migrated to the positions of putative monomers (Fig. 4E). Thus, the modification status of HRI caused by GSK-J4 was very similar to the reported one by heme depletion, although the connection of GSK-J4 action with heme metabolism has not been implicated. One of the major questions is why HepG2 did not respond to GSK-J4 for ATF4 induction as shown in Fig. 2A. Therefore, HRI was stably introduced into HepG2 and tested for ATF4 induction. ATF4 was significantly but not fully induced in the HepG2 + HRI cells compared to the parental one (Supplementary Fig. S1C), although no apparent correlation was observed between the HRI expression and the ATF4 induction levels in HCT116, HepG2 and SW13. This suggests that activation of HRI or other regulators in the HRI-ATF4 cascade by GSK-J4 is also involved in the cell type-specificity. Proteolytic processing of DELE1 mediates mitochondrial stress to the HRI activation^47^. We also observed that a short N-terminal portion of DELE1 was removed by GSK-J4 treatment (Supplementary Fig. S1D).

### Induction of apoptosis by GSK-J4 does not correlate with ATF4 or CHOP levels

**Figure 5:**
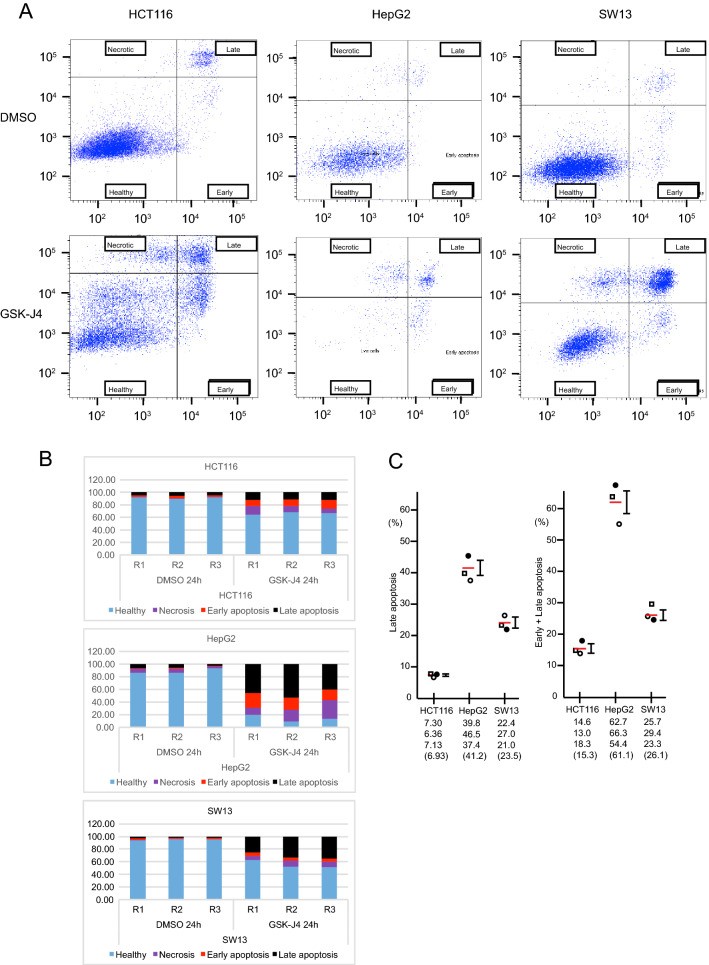
Apoptosis assay on GSK-J4 treated cell lines. (**A**) HCT116, HepG2, and SW13 cells were treated with 30 μM GSK-J4 for 20 h and stained with Annexin-V-Alexa Flour 647/Sytox-blue. Apoptosis was analyzed by flow cytometry in triplicate (N = 3) and representative data is shown. The horizontal and vertical axes indicate the Annexin-V and Sytox staining measures, respectively. The cell healthy/apoptotic states are denoted in red. (**B**) Quantitative representation of the cell healthy/apoptotic states with stacked bar charts. Results of three independent experiments (R1, R2, and R3) are presented. The vertical axis shows the percentage of cells with total cell numbers at 100%. N.A.: data not available. (**C**) Ratios of late or early + late apoptotic cells to total cells in each cell line are first calculated, and specific ratios were obtained by subtracting ratio(DMSO) from ratio(GSK-J4) and plotted as percentages. Values from each of the triplicate experiments are presented below and indicated by open square, closed circle and open circle, respectively, and the means are presented in parentheses and indicated by red lines. Standard error bars are included. Note that apoptosis induction is low in HCT116 and high in HepG2. Differences in percentages between HCT116 and HepG2 or SW13 are significant (p < 0.05). Also see Supplementary Fig. S1A,B for apoptosis induction by ATF4.

The ATF4-CHOP axis has been well characterized as a major pathway for ER stress-induced apoptosis^48–50^. GSK-J4 suppresses cell proliferation and causes apoptosis in mouse xenografts of human brainstem glioma cell lines SF8628^51^ and in a human acute myeloid leukemia cell line Kasumi-1^52^. We therefore analyzed GSK-J4-stimulated apoptosis with annexin V-Alexa Flour 647 staining (Fig. 5A). ATF4 and CHOP induction was high in HCT116, intermediate in SW13 and very low or negative in HepG2 (Fig. 2A,B). Interestingly, the extent of GSK-J4- induced apoptosis did not correlate with that of the ATF4 and CHOP induction. Ratios of the late or early + late apoptotic to total cells were low for HCT116, intermediate for SW13 and high for HepG2. (Fig. 5B,C). Therefore, the apoptosis induction by GSK-J4 cannot be simply due to increased levels of ATF4 and CHOP. To examine the ability of ATF4 to cause apoptosis in these cells, we tested the effect of CMV-driven stable ATF4 expression (Supplementary Fig. S2). HCT116 produced a high level of ATF4 but showed a moderate increase in the total apoptotic rate, whereas SW13 showed more extensive apoptosis with lower ATF4 expression. HCT116 is apparently more resistant to GSK-J4 and ATF4 expression. ATF4 activation by GSK-J4 may on the contrary promote cell survival, possibly by activation of autophagy^33,34^ and the apoptosis we observed appears to be a compromised phenomenon between these positive and negative effects on cell survival.

### UTX complementation down-regulates the ATF4 target genes in *UTX*-mutated HCT116 cells

**Figure 6:**
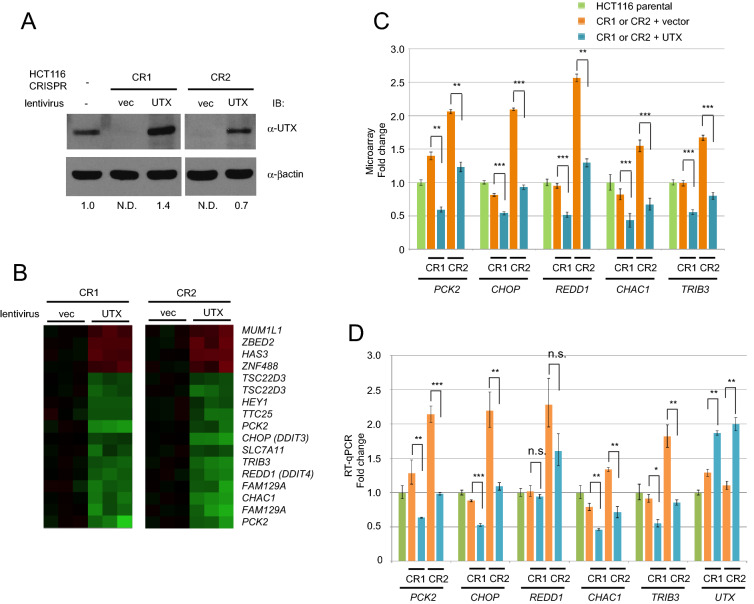
Gene expression profiling analysis of UTX-regulated genes using *UTX*-mutated and reconstituted cells. (**A**) * UTX*-mutated HCT116 cell lines (CR1 and CR2) were established by CRISPR gene editing and exogenous UTX was re-expressed using a lentivirus vector (vec: vector only; UTX: with exogenous UTX). UTX protein was detected by western blot analysis and the expression levels are shown under the lanes. Details of the targeted and mutated sequences are presented in Supplementary Fig. S3. (**B**) Gene expression profiling with microarray. Up- or down-regulated genes in the CR1 and CR2 clones are shown in the heatmap with a cut-off of 1.5-fold. Gene identities are shown on the right. (**C**) Expression levels of *PCK2, CHOP, REDD1, CHAC1* and *TRIB3* from the microarray analysis are shown in the bar charts. Data shows the mean of triplicates with S.E.M. and p-value (*< 0.05; **< 0.01; ***< 0.001). (**D**) RT-qPCR analysis. The same samples used for the microarray analysis were analyzed by RT-qPCR. n.s. indicates “not significant”. For microarray and RT-qPCR experiments, biological triplicates (N = 3) were processed.

The H3K27 demethylase UTX is one of the major targets of GSK-J4^21^. This raises a major question as to whether the effects of GSK-J4 (i.e. induction of ATF4, and its downstream target genes) are attributed to inhibition of UTX function. To determine this, we mutated *UTX* a pseudodiploid colorectal cell line HCT116 by CRISPR-Cas9 editing^53^. Two sites immediately downstream of the initiator ATG were targeted (Supplementary Fig. S3). Two cell lines CR1 and CR2 that have 2 and 56 nucleotide frame-shift deletions, respectively, were obtained and showed nearly undetectable UTX expression (Fig. 6A). Cell lines usually consist of a mixture of genetically and phenotypically heterogeneous cell populations and independently isolated clones could show distinct properties. Therefore, we compared the edited clones and their derivatives that have been reconstituted with stably re-expressed UTX. Reconstituted levels of exogenous UTX were nearly equivalent to the endogenous level in the parental HCT116 cells (Fig. 6A), which could minimize artifacts caused by overexpression. Global levels of the H3K4me2, H3K9me2 and H3K27me3 histone marks remained unchanged (Supplementary Fig. S4) similarly to the previous report^54^. Microarray gene expression profiling experiments were conducted to identify UTX target genes. Somewhat unexpectedly, a relatively small number of up- and down-regulated genes were identified between UTX edited and reconstituted HCT116 cells using a 1.5-fold change cut-off (Fig. 6B,C), which could be explained by the little changes in the global histone modifications. Remarkably, most of the down-regulated genes have been reported to be regulated by ATF4 (e.g. *PCK2*, *CHOP*, *CHAC1, TRIB3* and *REDD1*). *FAM129A* (*C1orf24*, *NIBAN*)^55^ and *SLC7A11/xCT*^56^ are also ER stress-inducible, yet less characterized, genes. Importantly, genes regulated by the UPR via ATF6α or inositol-requiring protein-1α (IRE1α) were not among the differentially regulated genes. These results were highly reproducible in a parallel RT-qPCR experiment (Fig. 6D). *UTX* mRNA levels were comparable between the parental (HCT116-parental) and CRISPR-edited cell lines (CR1 or CR2 + vector), suggesting that the frame-shifted *UTX* mRNAs generated by CRISPR editing were transcribed similarly to the wild type mRNA (Fig. 6D). Re-expression increased *UTX* mRNA levels by 1.5- to 2.0-fold. Further meta-analysis revealed significant association between higher *UTX* expression and lower expression of the ATF4 target genes in subsets of acute myeloid leukemia (AML) and gastric cancer patient specimens (Supplementary Fig. S5). Moreover, we previously showed that exogenous UTX expression in UTX-negative HeLa cells caused less colony formation^9^. Thus, even without the *UTX* mutations, anomalies affecting *UTX* expression levels such as haploinsufficiency and DNA methylation in the promoter region may be significant contributors to cancer formation^9^.

### UTX knockdown by shRNA also induces ATF4

To further examine the effects of UTX on ATF4, UTX knockdown experiment was performed. UTX was downregulated with shRNA stably expressed from a lentivirus vector in HCT116. Two independent shRNAs efficiently suppressed UTX protein expression (Supplementary Fig. S6A). In the absence of GSK-J4, the basal levels of ATF4 were moderately increased by the shRNAs (Supplementary Fig. S6A,B). In its presence, ATF4 levels were further increased, but the levels after induction did not reach that of the control (compare lanes 4 or 6 with lane 2). This might imply that suppression of UTX and GSK-J4 treatment are functionally redundant or, in other words, that regulation of ATF4 by GSK-J4 is at least partially due to UTX function.

## Discussion

Molecular mechanisms of cancer formation by loss of the tumor suppressor *UTX* have still remained poorly elucidated. We have sought clues by examining physiological effects of the specific KDM6 inhibitor GSK-J4 in epithelial cells. Here, we have shown that (1) the KDM6 inhibitor GSK-J4 specifically and potently increases the ATF4 protein level and the expression of its downstream target genes in a cell-type specific manner; (2) the ATF4 induction is not due to *ATF4* transcriptional or ATF4 post-translational regulation; (3) exogenous expression of HRI but not other ISR kinases efficiently restores the ATF4 induction in MEF lacking expression of all the four ISR kinases; (4) GSK-J4 induced apoptosis is partially dependent on the ATF4-CHOP axis; (5) UTX re-expression in UTX-mutated HCT116 specifically downregulates a set of the ATF4 target genes.

**Figure 7:**
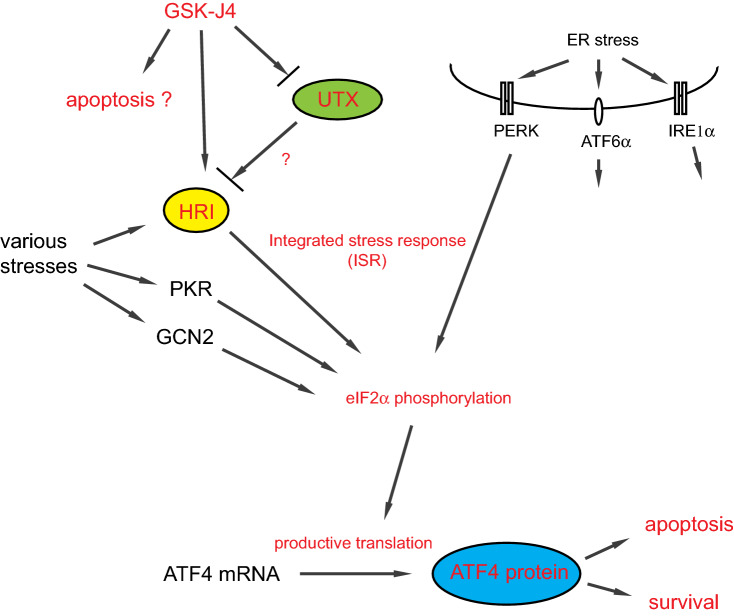
Schematic representation of GSK-J4 and UTX actions. UPR by ER stress and ISR by various stresses transduce signals downward to induce eIF2α phosphorylation and productive ATF4 translation. GSK-J4 induces eIF2α phosphorylation and ATF4 through HRI activation, which is either by suppression or independently of the UTX H3K27 catalytic activity. GSK-J4-induced ATF4 leads to apoptosis or cell survival in a context-dependent manner. Key molecules and regulatory events relevant to our findings are denoted in red.

Distinct from the ER stress response, four ISR kinases including PERK are activated by various stresses with differential yet overlapping spectra and phosphorylate eIF2α^42^ as summarized (Fig. 7). HRI is almost exclusively required for response to a mitochondrial oxidative phosphorylation uncoupler FCCP^43^ and to other mitochondrial stress inducers^47^. Mitochondrial stress activates a mitochondrial protease OMA1 that cleaves a mitochondrial protein DELE1, and the cleaved form of DELE1 accumulates in the cytoplasm and activates HRI by physically associating with HRI^47^. Since HRI is nearly indispensable for ATF4 activation by GSK-J4 (Fig. 4A), it is likely that GSK-J4 activates HRI by causing mitochondrial stress. Mechanisms of the HRI activation by GSK-J4 and suppression of UTX can be explained as follows. First, UTX demethylates a target protein and thereby induces mitochondrial stress in some way. This is possible, since an increasing number of non-histone target proteins of histone modifiers have been identified and a significant fraction of UTX is present in the cytoplasm^9^. Secondly, GSK-J4 induces mitochondrial stress irrelevant to UTX function. GSK-J4 is an α-KG antagonist which could affect integrity of the TCA cycle and, as a consequence, give mitochondrial stress. For instance, accumulation of succinate caused by *succinate dehydrogenase* (*SDH*) mutations results in inhibition of the α-KG-dependent reactions and the production of reactive oxygen species (ROS)^57^. Hydrogen peroxide, one of ROS, actually activates HRI^47^. Unexpectedly, we have consistently observed mere subtle activation of eIF2 α phosphorylation by GSK-J4 in HCT116 (Figs. 1B, 2B), while it was very high in MEF-4KO + HRI (Fig. 4B). Intriguing is a recent report that impairment of the heme biosynthesis pathway enhances eIF2α phosphorylation and also eIF5B protein levels, likely via HRI activation, and leads to induction of PD-L1 productive translation from the downstream initiation site^58^.

The ATF4-CHOP axis positively or negatively acts on the cell death-survival decision by transcriptionally regulating the numerous target genes. For instance, ATF4 and CHOP activate expression of the chaperon immunoglobulin heavy-chain binding protein (BIP or GRP78) and autophagy components and thereby could promote cell survival^33,34^, whereas CHOP efficiently induces apoptosis and suppresses tumor^47–49^. Overexpression of BIP or CHOP is frequently observed in various clinical cancer specimens and often correlates with higher cell malignancy, higher tumor grade and shorter patient survival^59^. When the feedback regulation fails to alleviate ER stress, cells are directed toward apoptosis through the intrinsic and extrinsic pathways^59–62^. As such, the physiological outcome of the ATF4-CHOP signaling appears to be dependent on their balance. We found that apoptosis does not correlate well with the ATF4-CHOP induction levels with the use of several epithelial cell lines (Fig. 5), which seemingly mirrors the aforementioned complexity.

UTX downregulated expression of the ATF4 target genes highly specifically, but the magnitudes of the downregulation were modest (1.5- to 2.5-fold) compared to those by GSK-J4 (compare Fig. 6 with Fig. 1). This discrepancy could be explained as follows: cell clones with less ATF4 inducibility were selected during the relatively long-term isolation period to escape potential toxic effects of the ATF4-CHOP axis; other GSK-J4-sensitive factors than UTX are also involved in the ATF4 induction. Identification of the direct targets of UTX is certainly the next step to understand this unique phenomenon. These future lines of investigation should also provide further direction on therapeutic avenues for treatment of cancer patient with GSK-J4 and related compounds.

## Materials and methods

### Cell culture

All cell lines except for HepG2 except and the gene-edited MEFs^43^ were obtained from ATCC (Manassas, VA, USA). HepG2 cell identity was estimated to be 100% by the Centre for Translational Research and Diagnostics of the National University of Singapore. All cell lines were cultured in high glucose DMEM (Nacalai, Kyoto, Japan) supplemented with 10% FBS, 2 mM glutamine, 100 U/ml penicillin and 100 μg/ml streptomycin (Thermo Fisher Scientific, Waltham, MA, USA). Primary renal proximal tubule epithelial cells (PRETEC) (ATCC, PCS-400-010) were cultured in the recommended media (ATCC, PCS-400-030 and PCS-400-040) and used within tenfold expansion in cell number. GSK-J4 (12073) and CPI-455 (22127) were obtained from Cayman Chemical (Ann Arbor, MI, USA). Tunicamycin (T-7765) was from Sigma-Aldrich (St Louis, MO, USA). Cycloheximide and actinomycin D were from FUJIFILM Wako (Osaka, Japan). The final DMSO concentration was adjusted to 0.2% in all experiments with inhibitor treatment.

### Plasmids

*UTX* cDNA was expressed as previously described^9^. *ATF4, HRI and DELE1* cDNAs were isolated from human fibroblasts (WI-38) by PCR and confirmed by sequencing. All the cDNAs were Flag-tagged (DELE1 at the carboxy-terminus and the others at the amino-terminus) and inserted into the lentivirus vector CSII-CMV-MCS-IRES2-puro^9^. Lentivirus vector plasmids to express shRNA against UTX (shUTX1:TRCN0000359260 and shUTX2:TRCN0000359261) were purchased from Sigma-Aldrich. The CRISPR-Cas9 lentiviral vector (lentiCRISPR v2) was a gift from Dr. Feng Zhang (Addgene plasmid #52961).

### cDNA expression and down-regulation by shRNA

Lentivirus preparation and transduction were previously described^63^. For experiments with exogenous UTX expression, 48 h after infection, the cells were selected with 4 µg/ml puromycin for a further 48 h and then maintained in the absence of puromycin for up to 4 days for analysis. For experiments with exogenous ATF4 expression and down-regulation with shRNA, the selection period was shortened to 24 h and the cells were analyzed within 2 days after puromycin removal to avoid toxic effects.

### Preparation of cell extracts and histones

Whole cell lysates for western blot analysis were prepared with M-PER (Thermo Fisher Scientific) supplemented with 150 mM NaCl, 1 mM DTT, 1 mM PMSF, cOmplete protease inhibitor cocktail (Sigma-Aldrich), 10 μM MG132 (Sigma-Aldrich) and phosphatase inhibitor cocktails 2 and 3 (Sigma-Aldrich) as previously described^63^. Cells were treated with 30 μM GSK-J4 unless otherwise indicated. Histones were prepared by an acid extraction method^64^.

### Antibodies and western blot analysis

Antibodies used for western blot analysis were α-UTX (GTX121246) and α-HIF-1α (GTX127309) from GeneTex (Irvine, CA, USA); α-ATF4 (D4B8), α-ATF6α (D4Z8V), α-CHOP (D46F1), α-PERK (C33E10), α-S51-phosphorylated eIF2α (D9G8), α-LC3A (D50G8), α-LC3B (D11) and α-ATG12 (D88H11) from Cell Signaling Technology (Danvers, MA, USA); α-HRI (A14119) from ABclonal Technology (Woburn, MA, USA); α-H3 (MABI0301), α-H3K4me3 (MABI0304), α-H3K9me2 (MABI0307), and α-H3K27me3 (MABI0323) from Medical & Biological Laboratories (Nagoya, Japan); α-Flag (α-DDK, clone OTI4C5) from OriGene (Rockville, MD, USA) and α-βactin (A5441) from Sigma-Aldrich. An appropriate α-DELE1 antibody has not been available at present^47^. Anti-mouse or anti-rabbit secondary antibodies (GE Healthcare, Buckinghamshire, UK) with Western Lightning Plus-ECL (PerkinElmer, Waltham, MA, USA) were used for detection. For quantification of bands, the western blot images were measured with ImageJ (National Institute of Health, USA) and, after normalization with the control ßactin, compared with a control band. Antibodies used for immunofluorescence are α-ATF4 (D4B8) and α-CHOP (L63F7).

### Immunofluorescence (IF) staining

Immunofluorescence staining was performed by fixation with 4% formaldehyde for 10 min, permeabilizing with PBS containing 0.3% Triton X-100 for 10 min, blocking with PBS supplemented with 0.3% Triton X-100 and 5% BSA for 30 min and incubating with the primary antibody in PBS with 0.3% Triton X-100 and 1% BSA for 2 h, all at room temperature. This was followed by incubation with a secondary antibody (Alexa Fluor 488, Thermo Fisher Scientific) for 1 h at room temperature and mounting with Prolong Gold antifade reagent containing DAPI (Thermo Fisher Scientific). Cell images were obtained with BZ-710 microscope system (KEYENCE, Osaka, Japan) equipped with PlanApo lambda × 20 and × 40 (Nikon, Tokyo, Japan) objective lenses. Image capture was performed under the same conditions for each staining with DAPI or antibodies.

### CRISPR-Cas9 gene editing

The *UTX* gene was mutagenized by CRISPR-Cas9 editing with transient transfection of the lentiCRISPR v2 vector^53,65^. Briefly, cells were transfected with the vector for 48 h and, subsequently, incubated with 4 µg/ml puromycin for the following 48 h. Cells were extensively dissociated by treatment with 0.25% trypsin and plated in 96-well plates. Single cell-derived colonies were isolated and analyzed by western blotting and genomic PCR followed by direct sequencing.

### RT-qPCR

RT-qPCR was performed as previously described^63^. Three biological replicates (N = 3) and two technical replicates were quantified for each condition, and means of the technical duplicates were used for statistical calculations. The ΔΔC_T_ method was used to calculate gene expression levels with normalization to *Peptidylprolyl isomerase A* (*PPIA*) mRNA. Normalized gene expression was further calculated against the control samples to show fold changes. Primer sequences are shown in Supplementary Table S1.

### Gene expression profiling with microarray

Total RNA was extracted from cell cultures in biological triplicates using RNeasy Mini kit (Qiagen) and RNA quality was assessed on 2100 Bioanalyzer (Agilent Technologies, Santa Clara, CA, USA) using the RNA 6000 Nano Chip kit (Agilent) for intact 18S and 28S ribosomal peaks with RNA Integrity Number (RIN) > 7.5. Four hundred ng of total RNA was reverse-transcribed into cDNA and in vitro transcribed into biotin-labeled cRNA using TotalPrep RNA Amplification Kit (Illumina, San Diego, CA, USA) according to manufacturer recommendations. Seven hundred fifty ng of the biotin-labeled cRNA from each sample was hybridized to HumanHT-12 v4.0 beadchip microarrays (Illumina) and scanned on BeadArray Reader (Illumina) at scan factor 1. Raw intensity values were background subtracted with the BeadStudio Data Analysis software (Illumina; https://www.illumina.com/Documents/products/datasheets/datasheet_beadstudio.pdf) and normalized using the cross-correlation method^66^. The version 3.1.3.0 we used has been discontinued. Differential gene expression was analyzed based on a fold change cutoff of 1.5 by comparison between HCT116 cell lines reconstituted with the wild type UTX-expressing vector and the empty vector. Wild type UTX-reconstituted HCT116 cells compared to the empty vector controls for both CRISPR clones CR1 and CR2.

### Apoptosis assays

To determine the rate of GSK-J4-induced apoptosis, Annexin V-Alexa Flour 647 and Sytox-blue nucleic acid staining (Thermo Fisher Scientific) was performed as previously described^67^. Briefly, for the experiments with GSK-J4 treatments, 100 000 cells were plated in a 12-well plate in triplicate and treated with GSK-J4 or DMSO for 20 h. Cells were then trypsinized and stained with Annexin V and Sytox in Annexin buffer (10 mM HEPES, pH 7.4, 140 mM NaCl, 2.5 mM CaCl_2_) for 15 min on ice in the dark followed by flow cytometry using a BD FACS Aria III (BD Biosciences, San Jose, CA). The status of the cell populations was defined by the distribution of the two markers; apoptotic cells at the early stages (Annexin V-positive and SYTOX-negative), necrotic cells (Annexin V-negative and SYTOX-positive), apoptotic cells at the late stages (Annexin V-positive and SYTOX-positive) and healthy cells (Annexin V-negative and SYTOX-negative).

### Meta-analysis of gene expression profiles of cancer specimens

Gene expression data for cancer specimens were obtained from the public database IST Online (http://ist.medisapiens.com/#phenoplot).

### Statistics

Unpaired two-tailed Student’s t-tests were utilized to calculate p-values (*: 0.01 < p < 0.05, **: 0.001 < p < 0.01 and ***: p < 0.001). Data are presented as mean ± standard error of the mean (S.E.M.) after normalization against the *PPIA* housekeeping control.

### Ethics declarations

Human or animal subjects were not directly used in this study.

## Supplementary Information

Supplementary Information.

## Data Availability

The gene expression profiling data has been deposited in the Gene Expression Omunibus (GEO, NCBI) with the Accession Number GSE127860.
