# Supplementary material for: A KDM6 inhibitor potently induces ATF4 and its target gene expression through HRI activation and by UTX inhibition
Source: Sci Rep. 2021 Feb 25;11:4538. doi: 10.1038/s41598-021-83857-y (PMC7907191; doi:10.1038/s41598-021-83857-y)
Supplement: Supplementary file 1 — Supplementary Information. [file 41598_2021_83857_MOESM1_ESM.pdf]

remove globally

Supplementary Information

A KDM6 inhibitor potently induces ATF4 and its target gene expression through HRI activation and partly by UTX inhibition

Shojiro Kitajima[1,2]*, Wendi Sun[1]*, Kian Leong Lee[1,3]*, Jolene Caifeng Ho[1], Seiichi Oyadomari[4], Takashi Okamoto[5], Hisao Masai[6], Lorenz Poellinger[1,7], and Hiroyuki Kato[1,5,6]***

[1]Cancer Science Institute of Singapore, National University of Singapore, 14 Medical Drive, Singapore 117599, Republic of Singapore; [2]The Institute for Advanced Biosciences, Keio University, Kakuganji 246-2, Mizukami, Tsuruoka, Yamagata 997-0052, Japan; [3]Cancer & Stem Cell Biology Program, Duke-NUS Medical School, 8 College Road, Singapore 169857, Republic of Singapore; [4]Institute of Advanced Medical Sciences, Tokushima University, Tokushima 770-8503, Japan; [5]Department of Molecular and Cellular Biology, Nagoya City University Graduate School of Medical Science, Mizuho-ku, Nagoya 467-860, Japan; [6]Genome Dynamics Project, Department of Basic Medical Sciences, Tokyo Metropolitan Institute of Medical Science, 2-1-6 Kamikitazawa, Setagaya, Tokyo 156-8506, Japan; [7]Department of Cell and Molecular Biology, Karolinska Institutet, SE-171 77 Stockholm, Sweden

Contents:

Table S1

Figure S1

Figure S2

Figure S3

Figure S4

Figure S5

Figure S6

Figure S7

Figure S8

Figure S9

Figure S10

Figure S11

Figure S12

Figure S13

Figure S14

Figure S15

Figure S16

Figure S17

Figure S18

Supplementary Table S1 Primer sequences used in RT-qPCR experiments

primer name	sequence
PCK2 F	ATCCACATCTGTGATGGAAC
PCK2 R	CGTCTTGCTCTCTACTCGTG
CHOP F	GGAGCTGGAAGCCTGGTATG
CHOP R	GCAGGGTCAAGAGTGGTGAA
REDD1 F	GAACTCCCACCCCAGATCGG
REDD1 R	CGAGGGTCAGCTGGAAGGTG
CHAC1 F	GTGGTGACGCTCCTTGAAGA
CHAC1 R	TTCAGGGCCTTGCTTACCTG
TRIB3 F	TGCCCTACAGGCACTGAGTA
TRIB3 R	GGTACCAGCCAGGACCTCA
UTX F	GCACGAAAAACAAGCGGAAAC
UTX R	TGAGGCGGATGGTAATGGAG
PPIA F	ACGGCGAGCCCTTGG
PPIA R	TTTCTGCTGTCTTTGGGACCT
ATF4 F	GCACATTCCTCGATTCCAGC
ATF4 R	GTGTCATCCAACGTGGTCAG

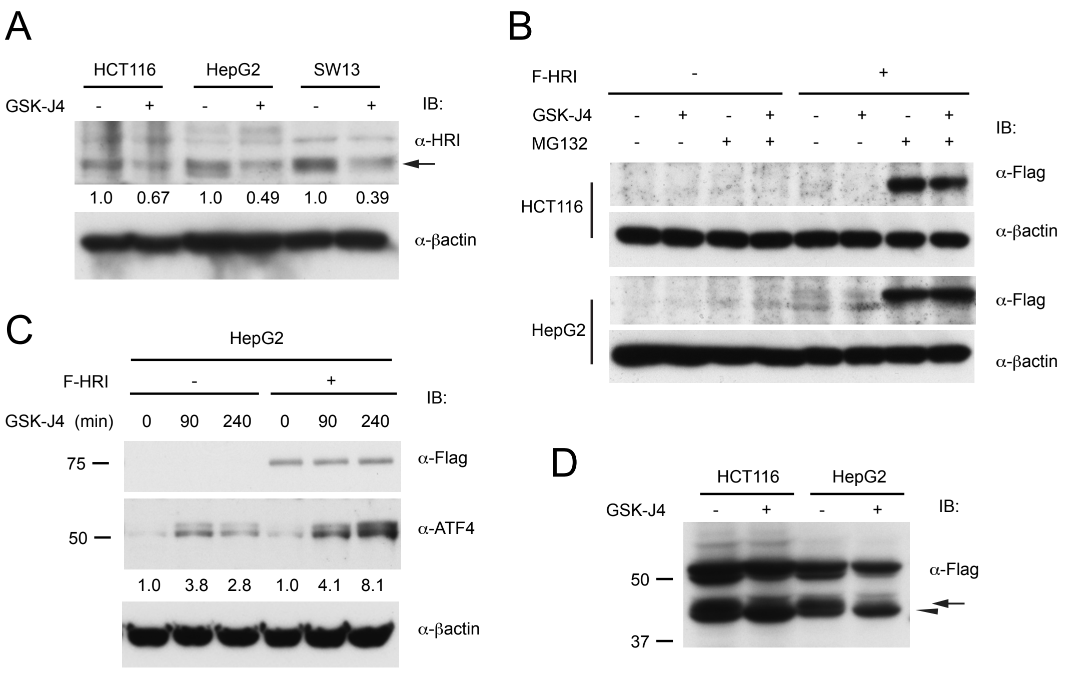

Supplementary Figure S1. HRI in human cell lines. *A,* Endogenous HRI (indicated by arrow) after GSK-J4 treatment of HCT116, HepG2 and SW13 for 240 min. Note that HRI levels decreased in all the three cell lines. *B,* Effect of treatment with GSK-J4 and/or MG132 for 20 h on stably expressed exogenous F-HRI. *C,* Effect of exogenous HRI expression on ATF4 induction. Exogenously expressed F-HRI in HepG2 significantly enhanced ATF4 induction. *D,* Effect of GSK-J4 treatment on DELE1 processing. DELE1 migrated at a slightly lower position after GSK-J4 treatment (arrowhead) than before (arrow).

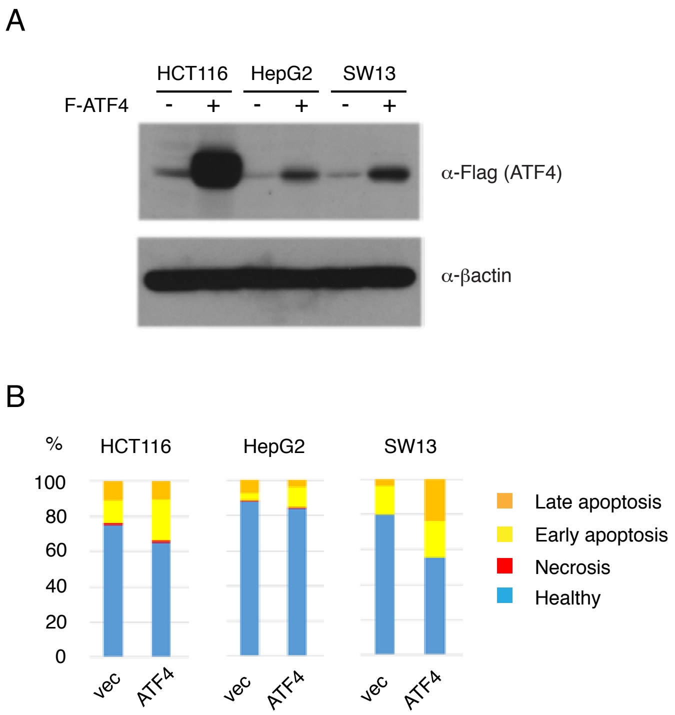

Supplementary Figure S2. Effect of ATF4 on apoptotic status. *A,* Stable expression of exogenous ATF4 in HCT116, HepG2 and SW13 cells. Flag-tagged ATF4 was detected with an anti-Flag antibody. *B,* Apoptosis analysis of ATF4-expressing cells. Cells were analyzed as in Fig. 5.

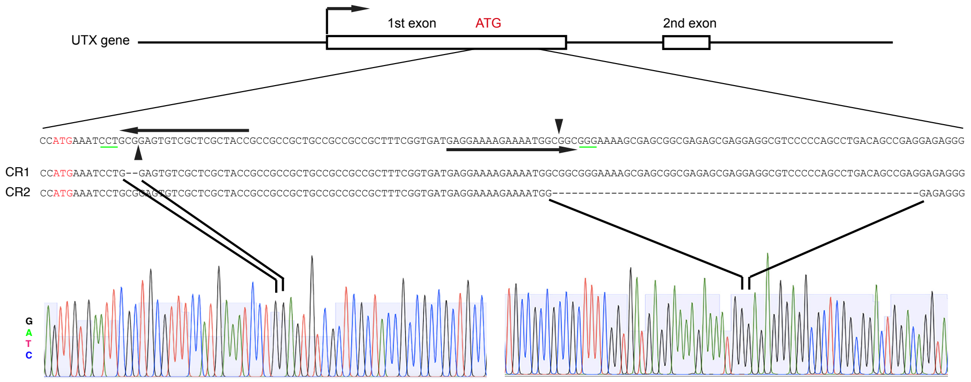

Supplementary Figure S3. UTX mutagenesis by CRISPR genome editing. Two positions in the 1st exon of *UTX* in HCT116 were targeted. Targeted sequences and PAM nucleotides are indicated by arrows and green underlines, respectively. Expected cleavage sites (indicated by arrowheads) are between +13 and +14, and +77 and +78 counting from the A of the initiator ATG (red) as +1. Two clones (CR1 and CR2) carrying 2 and 56 nucleotide frame-shift deletions, respectively, were isolated. DNA sequencing traces showing the mutations are attached in the lower panel.

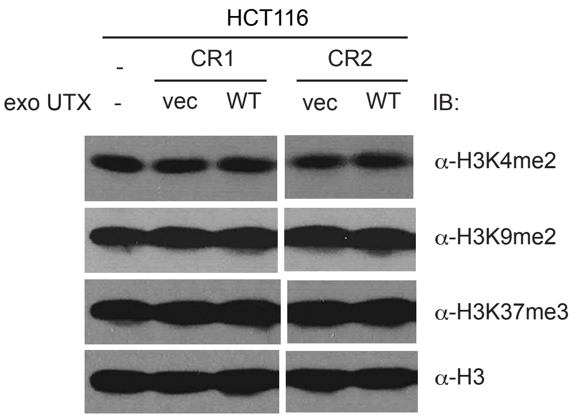

Supplementary Figure S4. Global histone modifications in CR1 and CR2. CR1 and CR2 clones used for gene expression profiling (Fig. 6A) were analyzed for histone H3 dimethy-K4 (H3K4me2), dimethyl-K9 (H3K9me2) and trimethyl-K27 (H3K27me3) methylation by western blot analysis. Note that no significant changes were detected.

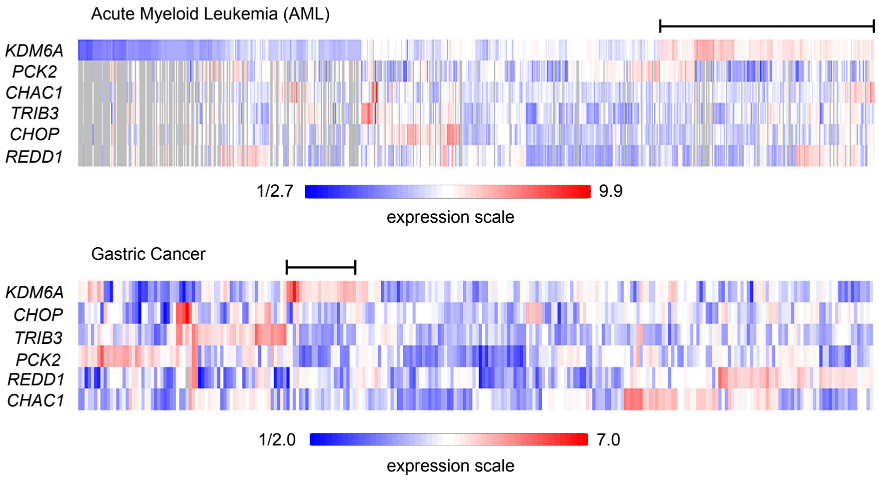

Supplementary Figure S5. Meta-analysis of gene expression association between *UTX* and ATF4 target genes in AML and gastric cancer. The color scale in the heatmaps corresponds to the mean gene expression of *UTX, PCK2, CHAC1, TRIB3, CHOP* or *REDD1* in AML and gastric cancer specimens. The colors range from darker blue (lower expression) through white (average expression) to brighter red (higher expression). Grey indicates that data is not available. The expression values range from 1/2.7 to 9.9-fold in AML and 1/2.0 to 7.0-fold in gastric cancer. There were a total of 1298 samples for AML and 372 for gastric cancer.

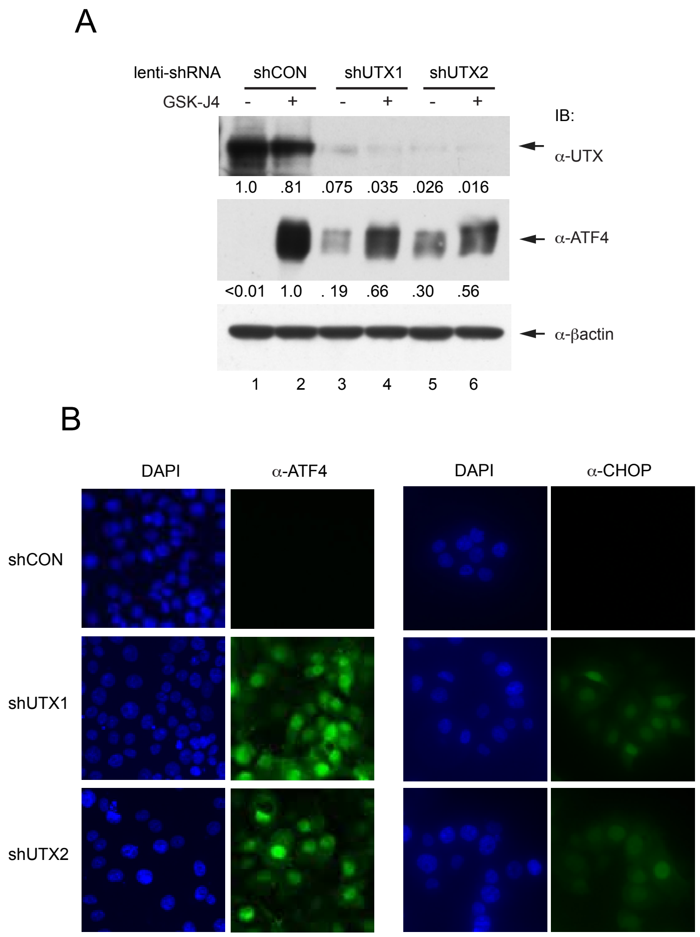

Supplementary Figure S6. Effects of UTX knockdown on ATF4 expression. *A,* Effect of UTX suppression. UTX was stably knocked down in HCT116 cells by lentiviruses expressing control (shCON), or shRNA against UTX (shUTX1 or shUTX2) and the cells were cultured in the absence or presence of 30 μM GSK-J4 for 20 h. UTX (upper panel) and ATF4 (middle panel) expression was analyzed by western blotting. Protein expression values of the quantified UTX and ATF4 bands are shown under the lanes. *B,* IF staining. The knocked down cells were analyzed by IF staining with antibodies against ATF or CHOP in the absence of GSK-J4.

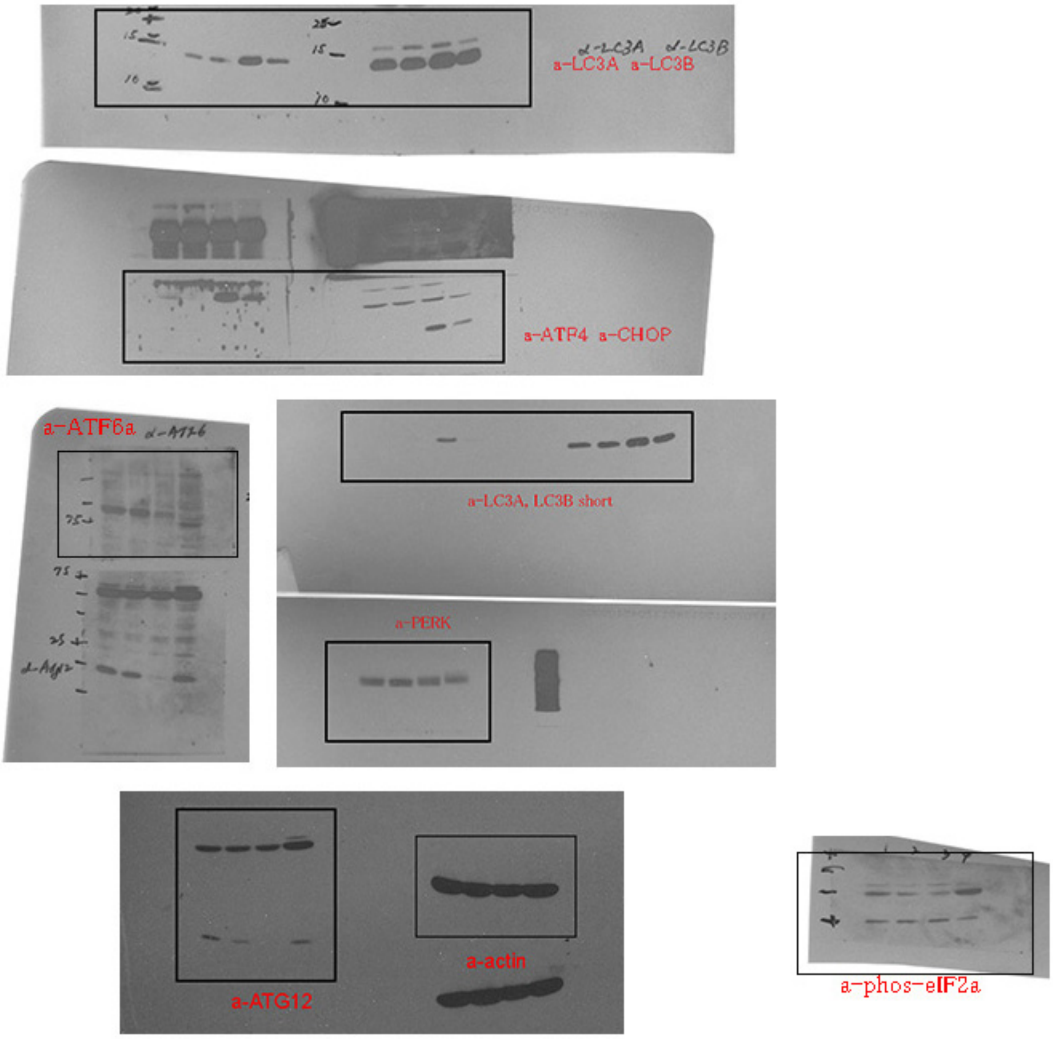

Supplementary Figure S7. Uncropped blots for Figure 1B. Cropped areas are indicated by square and red notes.

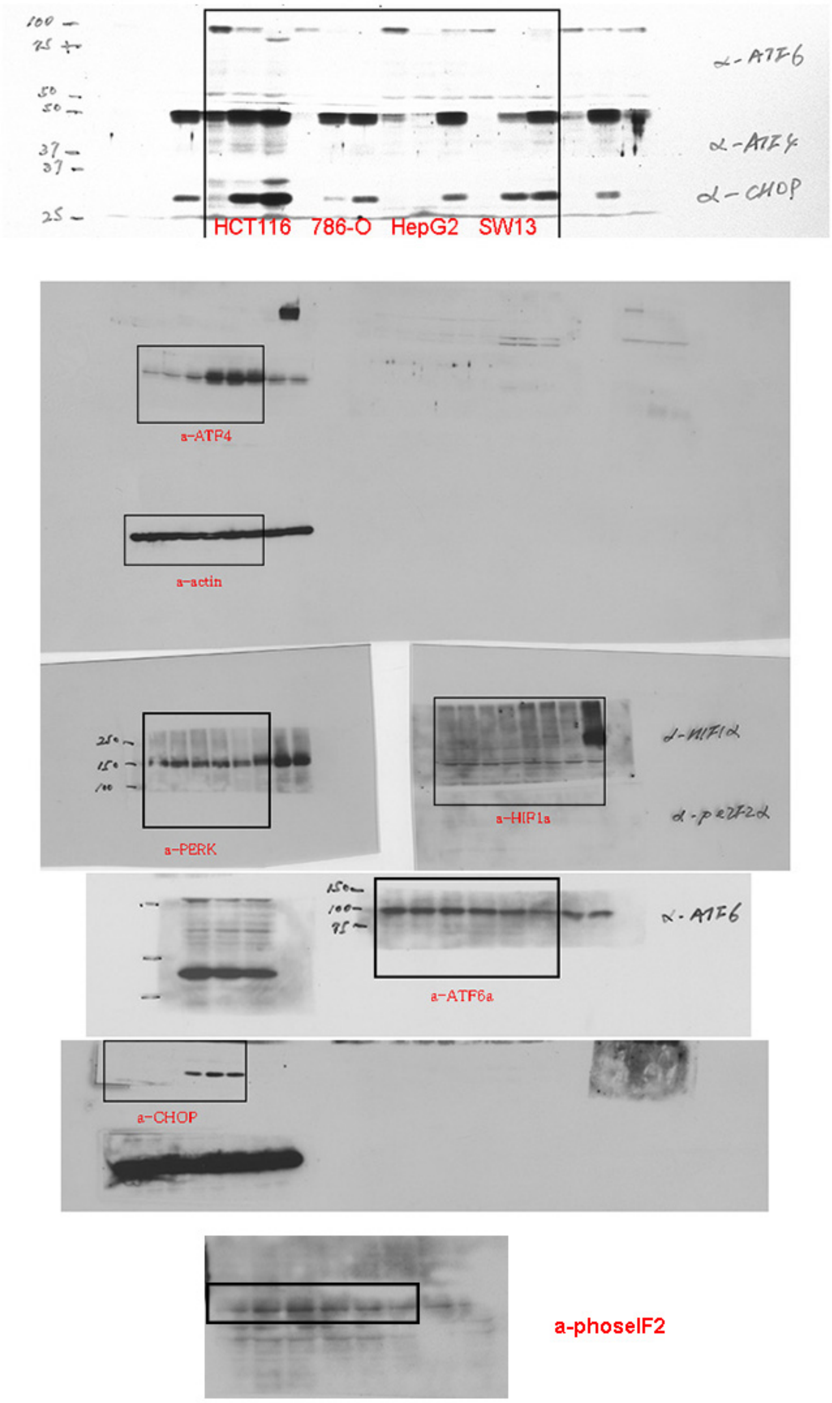

Supplementary Figure S8. Uncropped blots for Figure 2A,B.

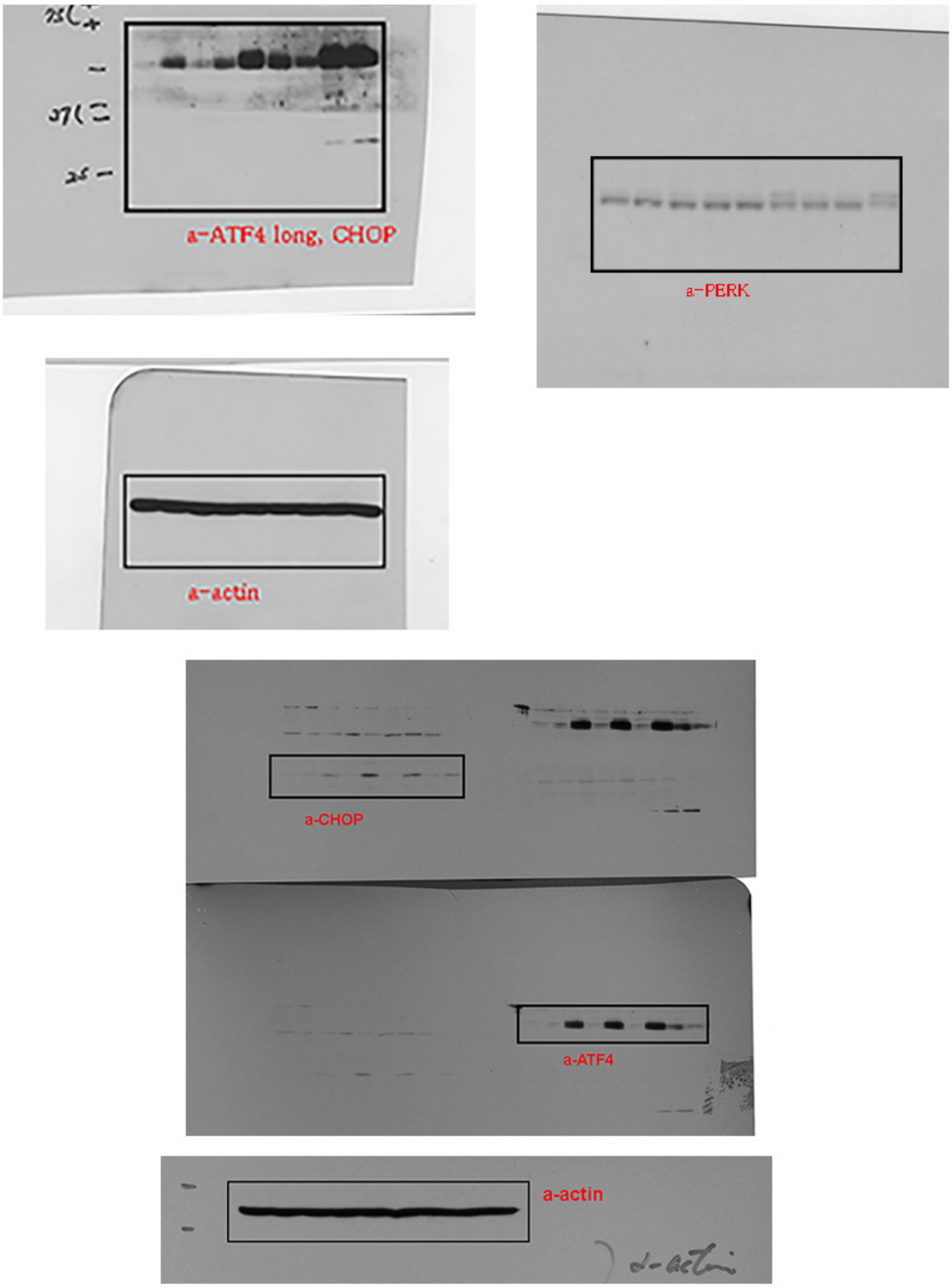

Supplementary Figure S9. Uncropped blots for Figure 2C.

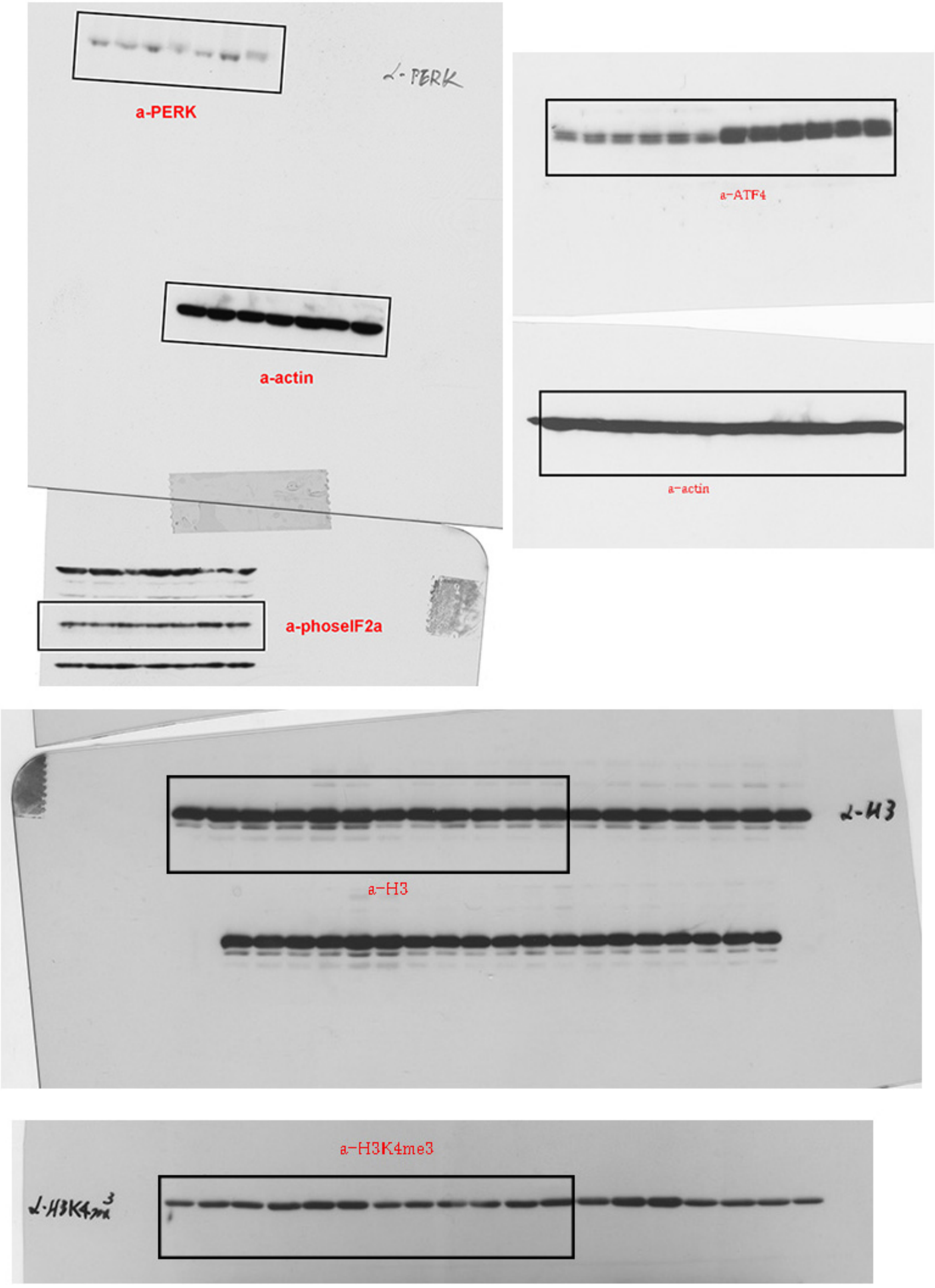

Supplementary Figure S10. Uncropped blots for Figure 2D,E.

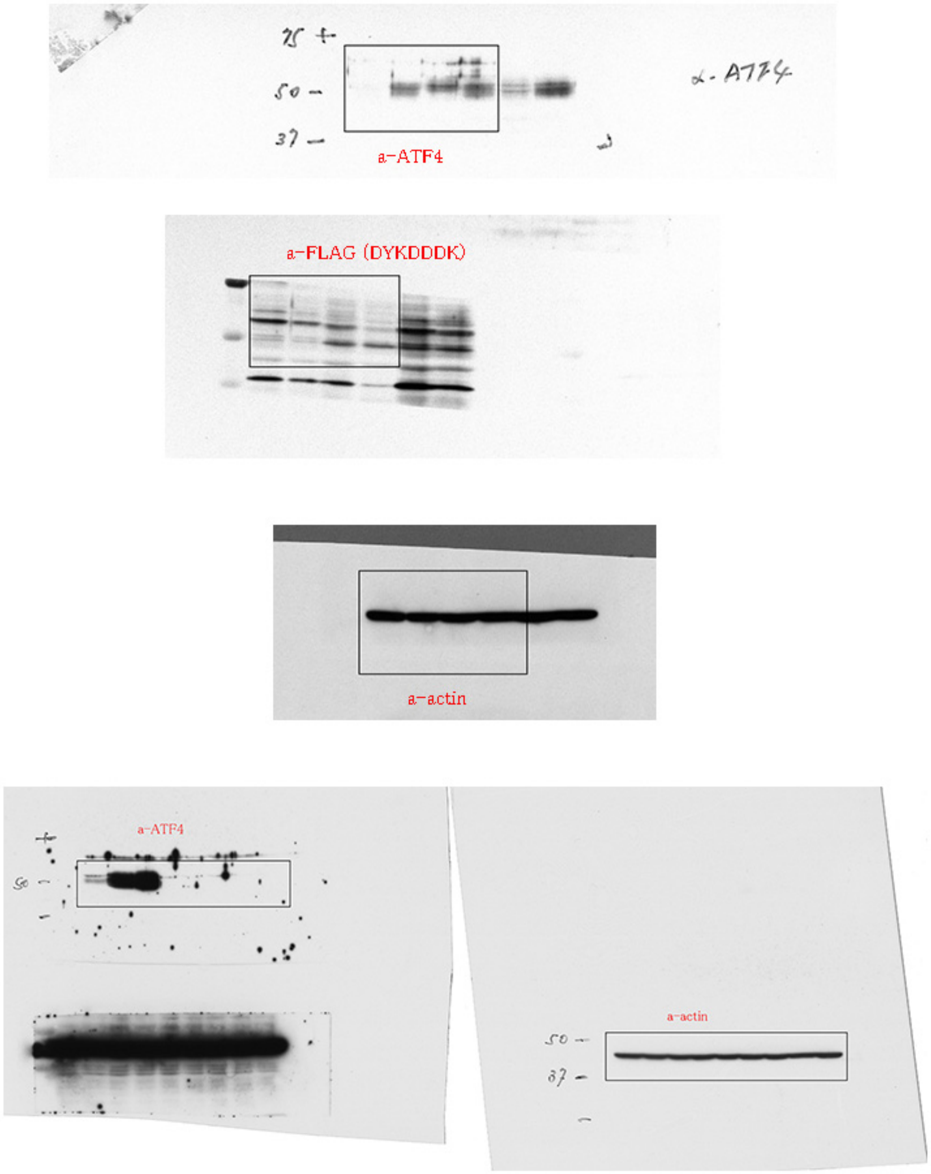

Supplementary Figure S11. Uncropped blots for Figure 3C,D.

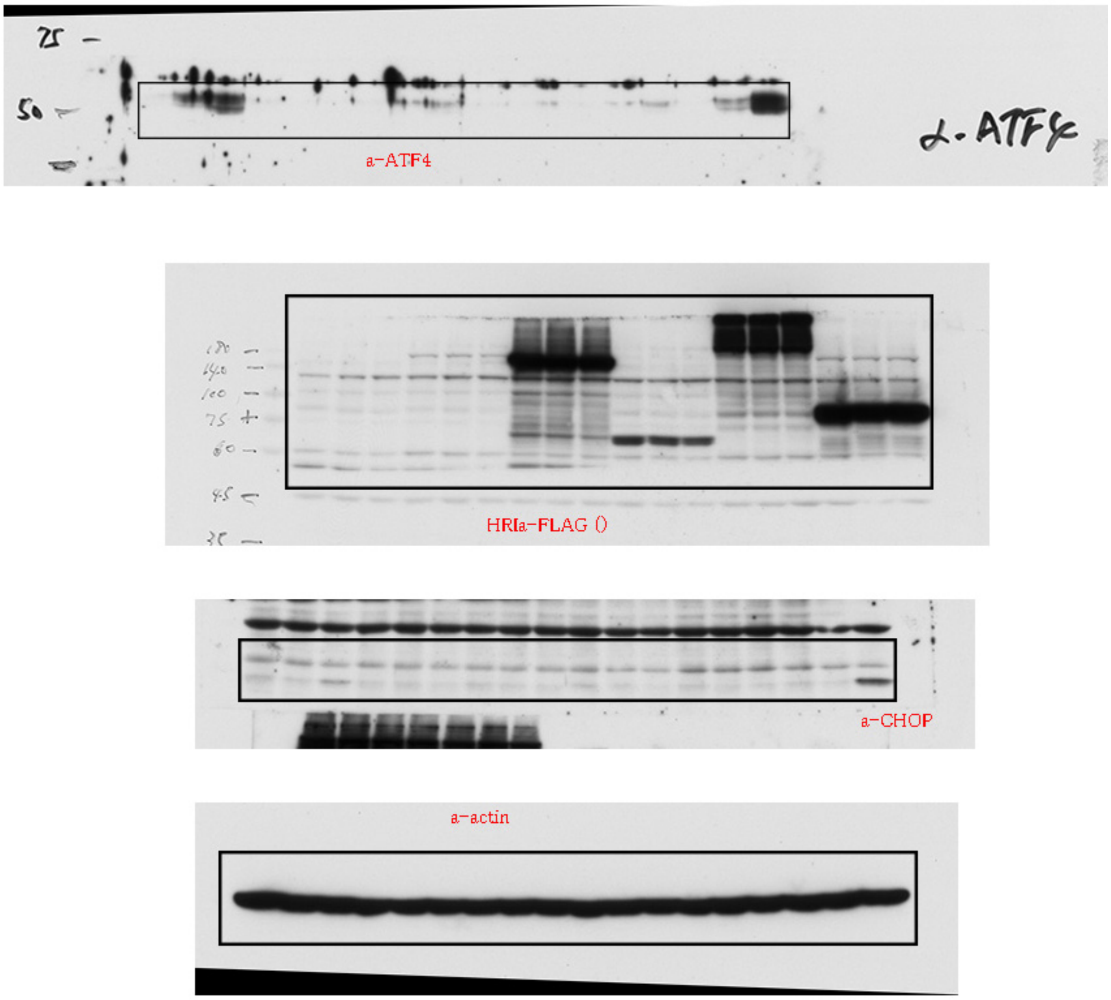

Supplementary Figure S12. Uncropped blots for Figure 4A.

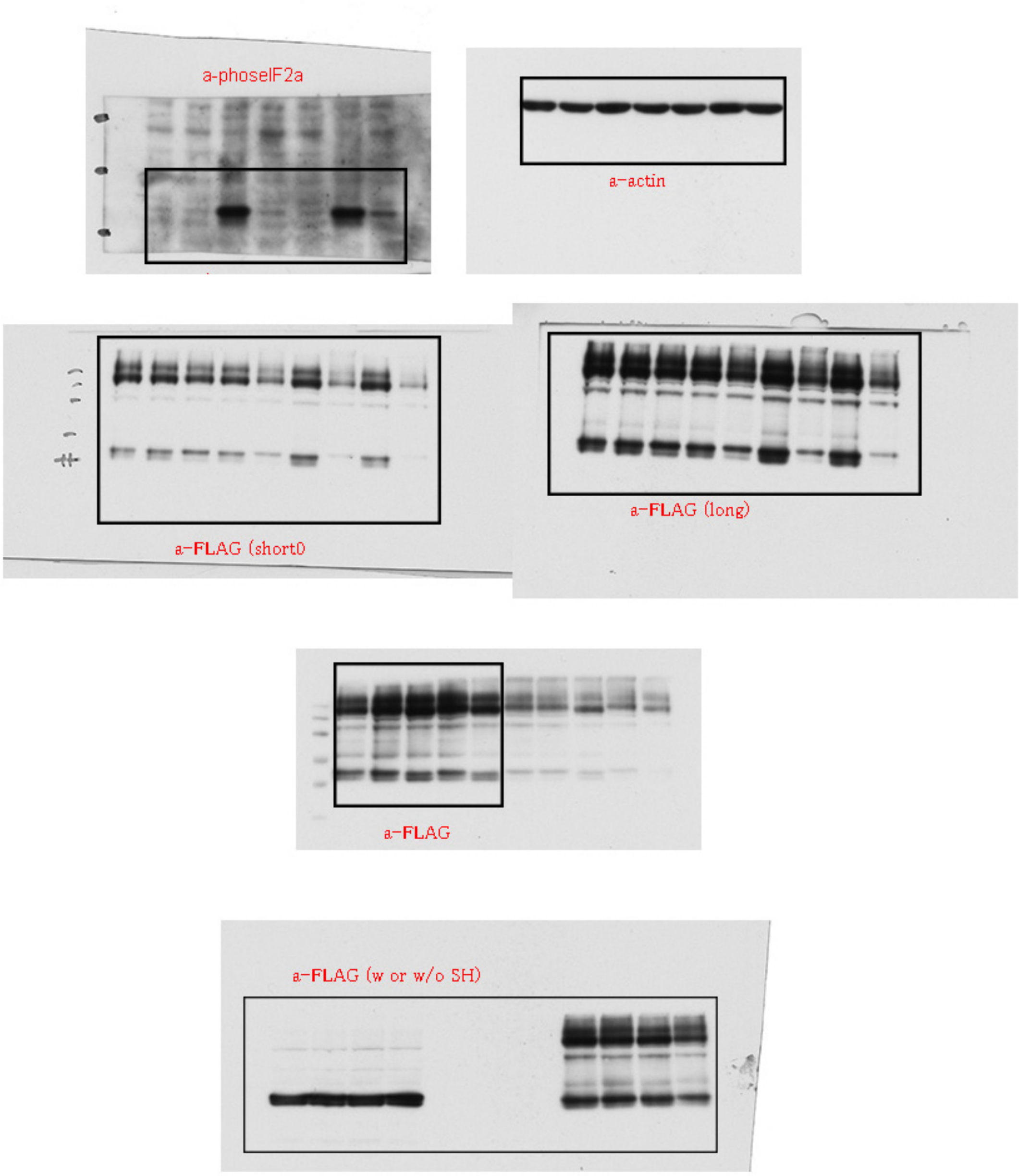

Supplementary Figure S13. Uncropped blots for Figure 4B-E.

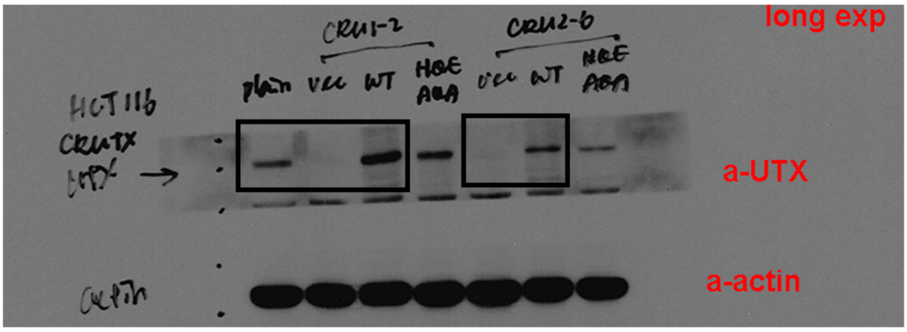

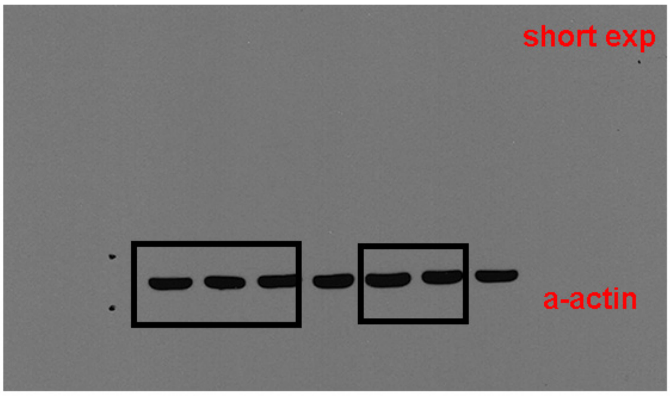

Supplementary Figure S14. Uncropped blots for Figure 6A.

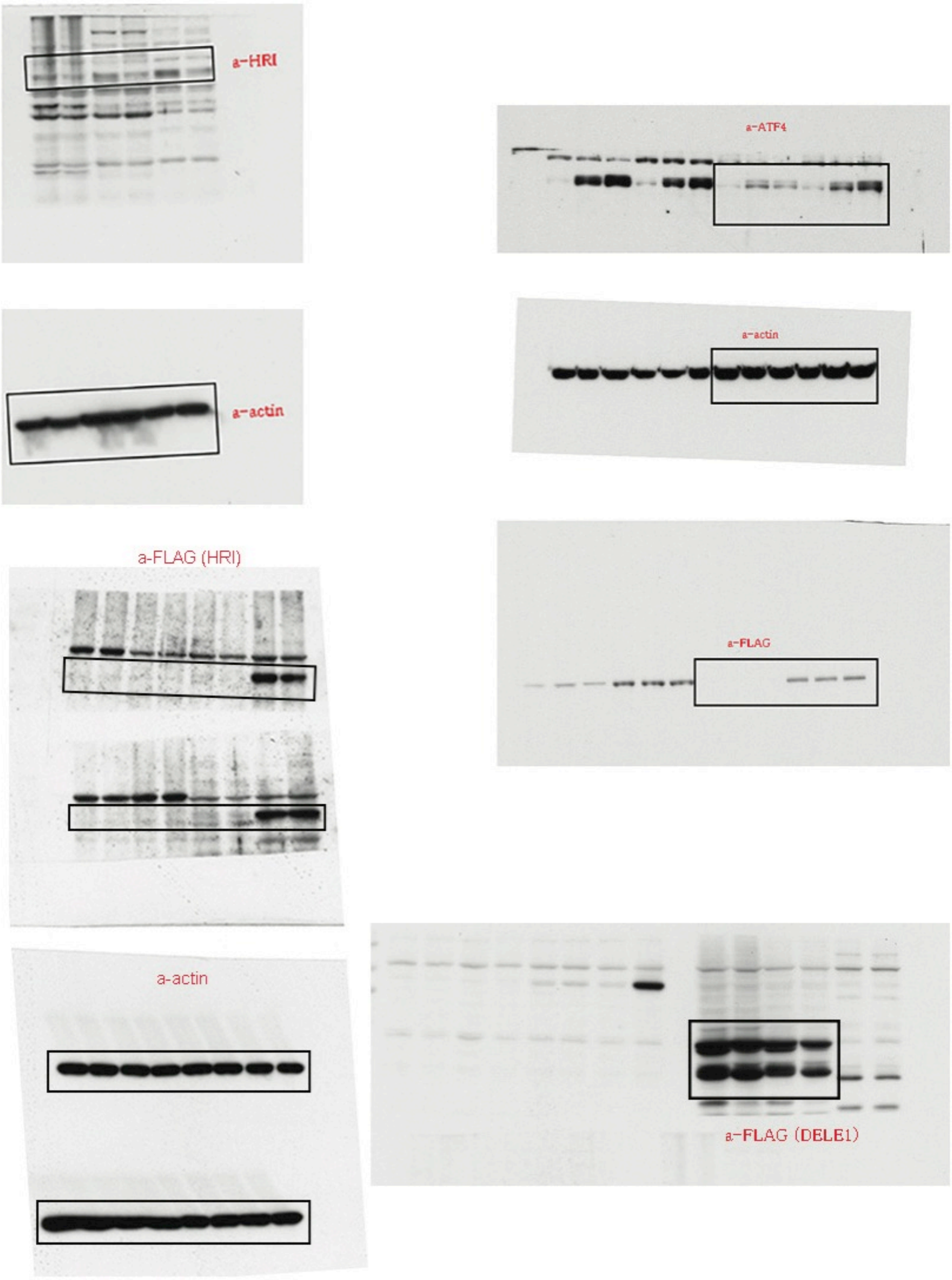

Supplementary Figure S15. Uncropped blots for Supplementary Figure S1.

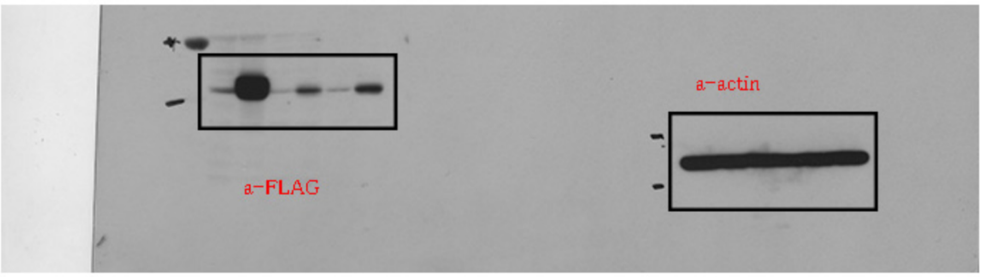

Supplementary Figure S16. Uncropped blots for Supplementary Figure S2A.

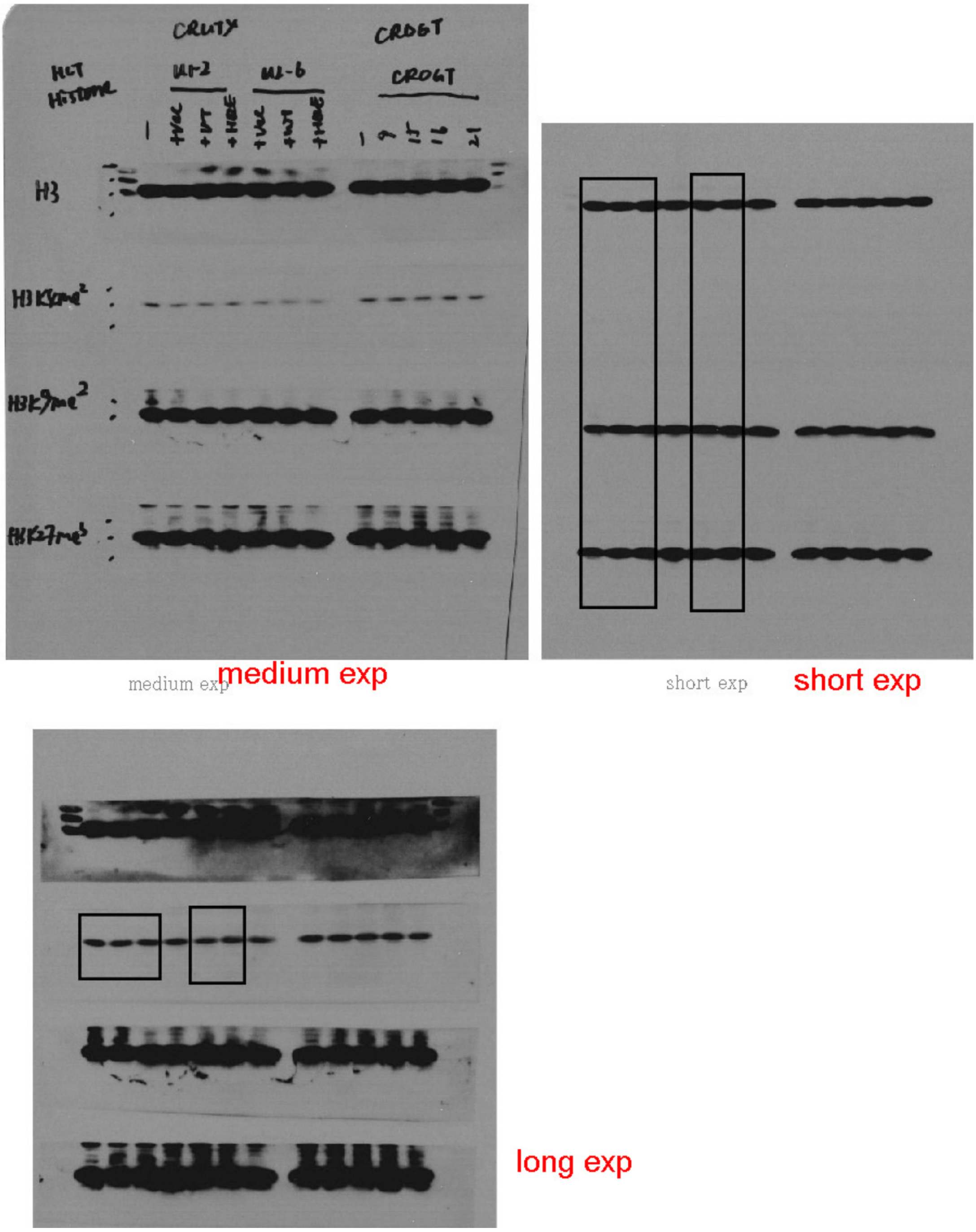

Supplementary Figure S17. Uncropped blots for Supplementary Figure S4.

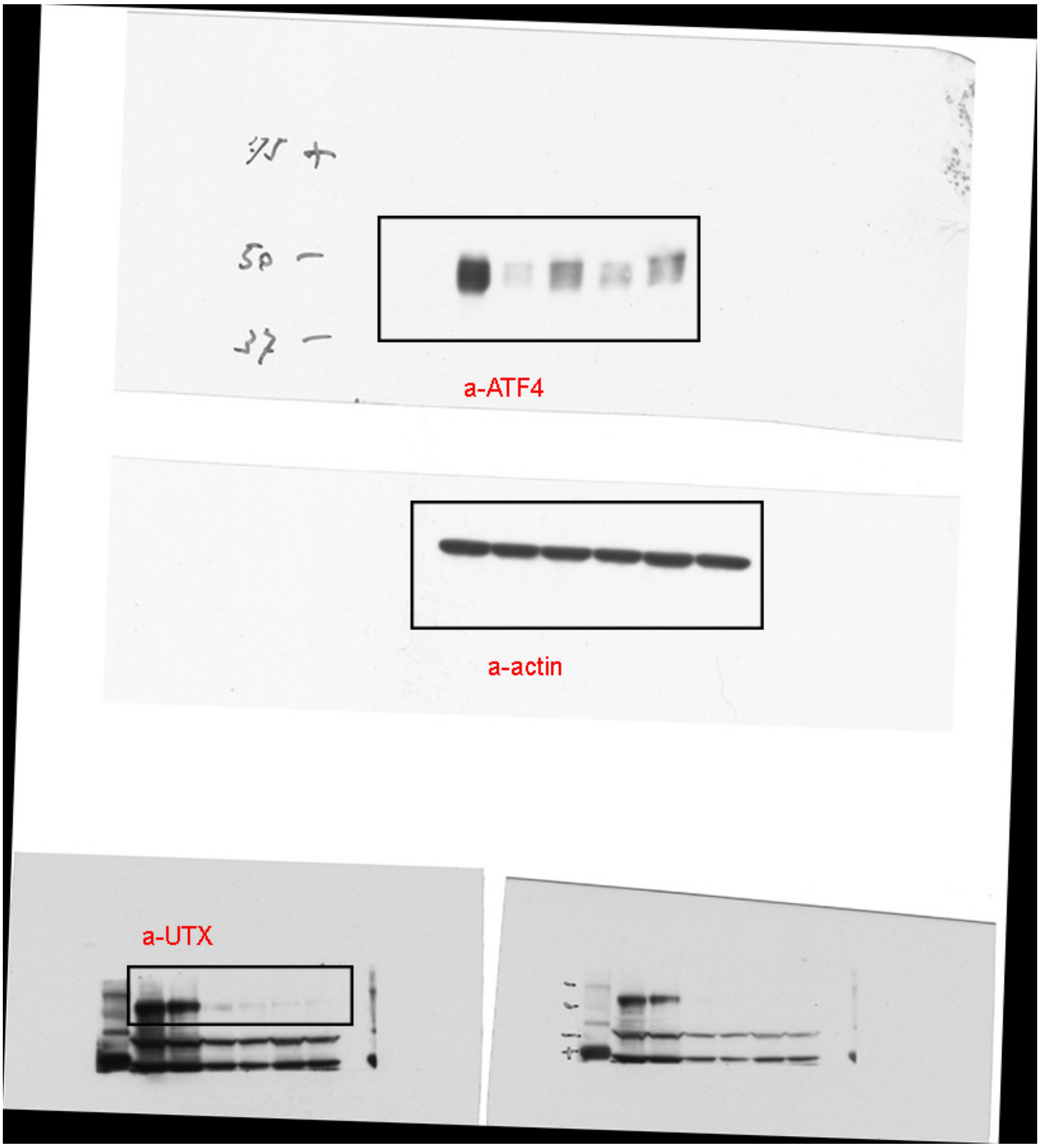

Supplementary Figure S18. Uncropped blots for Supplementary Figure S6.
